# Functional classification of DNA variants by hybrid minigenes: Identification of 30 spliceogenic variants of *BRCA2* exons 17 and 18

**DOI:** 10.1371/journal.pgen.1006691

**Published:** 2017-03-24

**Authors:** Eugenia Fraile-Bethencourt, Beatriz Díez-Gómez, Valeria Velásquez-Zapata, Alberto Acedo, David J. Sanz, Eladio A. Velasco

**Affiliations:** Splicing and genetic susceptibility to cancer, Instituto de Biología y Genética Molecular (CSIC-UVa), Valladolid, Spain; Cleveland Clinic Genomic Medicine Institute, UNITED STATES

## Abstract

Mutation screening of the breast cancer genes *BRCA1* and *BRCA2* identifies a large fraction of variants of uncertain clinical significance (VUS) whose functional and clinical interpretations pose a challenge for genomic medicine. Likewise, an increasing amount of evidence indicates that genetic variants can have deleterious effects on pre-mRNA splicing. Our goal was to investigate the impact on splicing of a set of reported variants of *BRCA2* exons 17 and 18 to assess their role in hereditary breast cancer and to identify critical regulatory elements that may constitute hotspots for spliceogenic variants. A splicing reporter minigene with *BRCA2* exons 14 to-20 (MGBR2_ex14-20) was constructed in the pSAD vector. Fifty-two candidate variants were selected with splicing prediction programs, introduced in MGBR2_ex14-20 by site-directed mutagenesis and assayed in triplicate in MCF-7 cells. Wild type MGBR2_ex14-20 produced a stable transcript of the expected size (1,806 nucleotides) and structure (V1-[*BRCA2*_exons_14–20]–V2). Functional mapping by microdeletions revealed essential sequences for exon recognition on the 3’ end of exon 17 (c.7944-7973) and the 5’ end of exon 18 (c.7979-7988, c.7999-8013). Thirty out of the 52 selected variants induced anomalous splicing in minigene assays with >16 different aberrant transcripts, where exon skipping was the most common event. A wide range of splicing motifs were affected including the canonical splice sites (15 variants), novel alternative sites (3 variants), the polypyrimidine tract (3 variants) and enhancers/silencers (9 variants). According to the guidelines of the American College of Medical Genetics and Genomics (ACMG), 20 variants could be classified as pathogenic (c.7806-2A>G, c.7806-1G>A, c.7806-1G>T, c.7806-1_7806-2dup, c.7976+1G>A, c.7977-3_7978del, c.7977-2A>T, c.7977-1G>T, c.7977-1G>C, c.8009C>A, c.8331+1G>T and c.8331+2T>C) or likely pathogenic (c.7806-9T>G, c.7976G>C, c.7976G>A, c.7977-7C>G, c.7985C>G, c.8023A>G, c.8035G>T and c.8331G>A), accounting for 30.8% of all pathogenic/likely pathogenic variants of exons 17–18 at the BRCA Share database. The remaining 8 variants (c.7975A>G, c.7977-6T>G, c.7988A>T, c.7992T>A, c.8007A>G, c.8009C>T, c.8009C>G, and c.8072C>T) induced partial splicing anomalies with important ratios of the full-length transcript (≥70%), so that they remained classified as VUS. Aberrant splicing is therefore especially prevalent in *BRCA2* exons 17 and 18 due to the presence of active ESEs involved in exon recognition. Splicing functional assays with minigenes are a valuable strategy for the initial characterization of the splicing outcomes and the subsequent clinical interpretation of variants of any disease-gene, although these results should be checked, whenever possible, against patient RNA.

## Author summary

A significant proportion of disease-causing mutations of inherited disorders impair splicing. Massive sequencing projects of genetic diseases generate thousands of sequence variations that require functional and clinical interpretations. We have shown that splicing reporter minigenes of the breast cancer genes *BRCA1* and *BRCA2* are useful tools to functionally test DNA variants. In this work, we have constructed a 7-exon *BRCA2* minigene (exons 14 to 20) where we mapped critical splicing regulatory sequences and tested 52 selected variants of exons 17 and 18 detected in breast cancer patients. We finely located three DNA segments on both exons that presumably contain splicing enhancer sequences. We observed that a total of 30 variants of any type disrupted the splicing patterns and, given the severity of their outcomes, we classified 20 of them as pathogenic or likely pathogenic. We also showed that a wide range of splicing elements were affected including canonical and novel 5’ and 3’ splice sites, the polypyrimidine tract and enhancer and silencer sequences. We concluded that splicing aberrations are frequent in Hereditary Breast and Ovarian Cancer and that minigenes are valuable tools to functionally classify DNA variants of any human disease gene under the splicing viewpoint.

## Introduction

Germline pathogenic variants in the tumor suppressor genes *BRCA1* (MIM# 113705) and *BRCA2* (MIM# 600185) are associated with increased risk of breast and ovarian cancer [1,2], and account for about 16% of the familial risk for breast cancer [3]. More than 25 breast cancer susceptibility genes have been identified so far, most of which play a role in the DNA repair pathway linked to *BRCA1* and *BRCA2* [4]. Additionally, a vast number of SNPs have been associated with breast cancer risk [5,6], increasing the complexity of the genetic landscape of Hereditary Breast/Ovarian Cancer (HBOC). Moreover, according to the BRCA Share Database (http://www.umd.be/BRCA2/; last accessed date, April 2016), more than 2700 different sequence variations have been reported at the *BRCA2* gene, ~30% of which are causal. A large proportion of the recorded pathogenic variants truncate the BRCA2 protein (nonsense and frameshift). However, up to 20% of *BRCA1/2* tests report variants of uncertain clinical significance (VUS) [7]. These pose a challenge in genetic counselling as VUS-carrier families are usually considered as negative (undetermined) so they cannot benefit from prevention protocols [8].

In fact, other factors must be involved in the pathogenesis of genetic disorders since gene expression is regulated by a wide range of cis-regulatory sequences that control it, as for example, transcription initiation (promoter) [9], pre-mRNA splicing [10] or post-transcriptional regulation and mRNA stability (3’UTR) [11]. It is therefore expected that point mutations in those motifs can be correlated with gene expression alterations and disease. Interestingly, nearly 90% of disease-associated SNPs are placed outside protein-coding regions (45% intronic, 43% intergenic), suggesting a relevant role of the non-coding sequence variations [12]. Splicing is a central process of gene expression whereby introns are excised and exons are joined sequentially. It has been calculated that >90% of mammalian genes undergo alternative splicing which is controlled by a dense array of diverse cis-acting elements and splicing factors [10,13]. According to GENCODE (V.24, http://www.gencodegenes.org/stats/current.html), the average number of protein coding transcripts per gene is ~4. In this regard, it has been recently reported the existence of 24 naturally occurring alternative splicing events of the *BRCA2* gene [14]. Alternative splicing not only allows transcriptome and proteome diversity, but it also regulates important processes such as embryonic development or cell differentiation. Exon recognition requires specific signals at the 5’ and 3’ splice sites, the polypyrimidine tract, the branch point, and supplementary sequences referred to as Exonic Splicing Enhancers (ESE) and Silencers (ESS) [15]. Interestingly, an unexpectedly large fraction of variants can actually disrupt pre-mRNA processing [16,17]. Remarkably, aberrant splicing is common in cancer so that it can be considered a hallmark of this disease [18]. We previously estimated that a significant proportion of likely pathogenic *BRCA1/2* variants (33.9%) from 14 exons would impair splicing [19].

The most suitable method to identify splicing aberrations is based on the study of patient RNA of the affected tissue although this sort of sample is not always available [20,21]. Nevertheless, direct RNA analysis of several disease genes, including *NF1* (MIM# 613113) and *BRCA1/2*, has proven to be a highly sensitive method to identify splicing anomalies [22,23]. Minigene-based technologies have become alternative approaches to primarily test whether a specific DNA variant affects splicing, especially when patient samples cannot be collected. In a recent report we presented the new splicing reporter plasmid pSAD (Patent P201231427-CSIC) that constituted the backbone of the largest *BRCA2* minigene ever reported with 9 exons (19 to 27) [24]. This construct allowed the analysis of 40 variants spread throughout these exons and flanking introns, demonstrating its capability for the successful identification and characterization of *spliceogenic* variants. Moreover, up to date, we have examined the impact on splicing of 112 different DNA variants by minigene assays, 51 of which induced aberrant splicing patterns [19,24,25].

The intronic GT dinucleotide in positions +1 and +2 is the most conserved element of the donor splice signal. However, in a small fraction of the donor sites (<1%), GT is replaced by GC that are rather located in alternatively spliced introns [26,27]. Recognition of a GC donor site at the *BTK* gene was associated with splicing enhancers for SR proteins 9G8, Tra2β and SC35 [27]. The *BRCA2* intron 17 has also a 5’ GC motif and there have been identified minor natural alternative splicing isoforms Δ18 (exon 18 skipping) and Δ17,18 (exons 17+18 skipping) [14,28]. To study the regulatory mechanisms of both exons and how DNA variants affect this process we constructed a large minigene with exons 14 to 20 in the pSAD plasmid (MGBR2_ex14-20) to keep the genomic context. Splicing regulatory elements were searched by functional microdeletion mapping. Finally, 52 variants detected in HBOC patients were selected and assayed in the minigene MGBR2_ex14-20.

## Results

**Fig 1:**
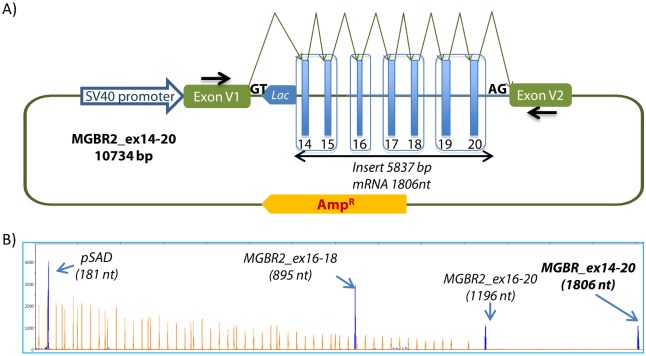
Structure and functional analysis of the minigene MGBR2_ex14-20. A) Structure of the minigene MGBR2_ex14-20 (slashes indicate shortened introns): [IVS14 (328 pb)–EX14 (428 pb)–IVS14 (1139 pb)—EX15 (182 pb)–IVS15 (358 pb) // IVS15 (333 pb)–EX16 (188 pb)–IVS16 (234 pb) // IVS16 (181 pb)–EX17 (171 pb)–IVS17 (485 pb)–EX18 (355 pb)–IVS18 (314 pb) // IVS18 (235 pb)–EX19 (156 pb)–IVS19 (398 pb)–EX20 (145 pb)–IVS20 (207 pb)]. Boxes highlight the 4 different cloning steps of this minigene. The expected splicing reactions in eukaryotic cells are indicated by arrows. B) Splicing functional analysis of the empty vector pSAD and the final (14–20) and intermediate (16–18, 16–20) constructs in MCF-7 cells. The four electropherograms were overlaid. cDNAs were amplified with vector exon specific primers SD6-PSPL3_RTFW and RTpSAD-RV (arrows within vector exons V1 and V2 above). Full-length transcripts are shown as blue peaks and the Genescan Liz-1200 size standard is shown as orange/faint peaks. Fragment sizes (bp) and relative fluorescent units are indicated on the x- and y-axes, respectively.

The minigene MGBR2_ex14-20 (10,734 bp) was built as indicated in materials and methods. The insert (5,837 bp) corresponds to a genomic region of 16,762 bp of the *BRCA2* gene (Fig 1A). To validate it, we transfected the final (14–20) and the intermediate constructs (17–18, 16–18 and 16–20) into MCF-7 cells, we isolated the RNA and performed the RT-PCR with specific primers at the vector-exons. Fig 1B shows the transcripts generated by the pSAD vector, two intermediate (16–18 and 16–20) and the final constructs of this minigene. MGBR2_ex14-20 produced a full-length transcript of the expected size (1,806 nucleotides—nt-) and structure (V1-*BRCA2* exons 14 to 20-V2) without any anomalies, so this minigene was ready for regulatory studies and variant analysis. The switch of the GC site of exon 17 to a strong canonical GT site (artificial variant c.7976+2C>T; NNSplice = 1.0) rendered the same canonical transcript without any anomalies (9 independent assays), like the GC counterpart (S1 Fig), that was confirmed by sequencing. Finally, exons 12 and 13 were also cloned in two independent stages but splicing reactions did not produce the expected canonical transcripts so they were ruled out.

### Mapping of exonic and intronic splicing regulatory sequences

**Fig 2:**
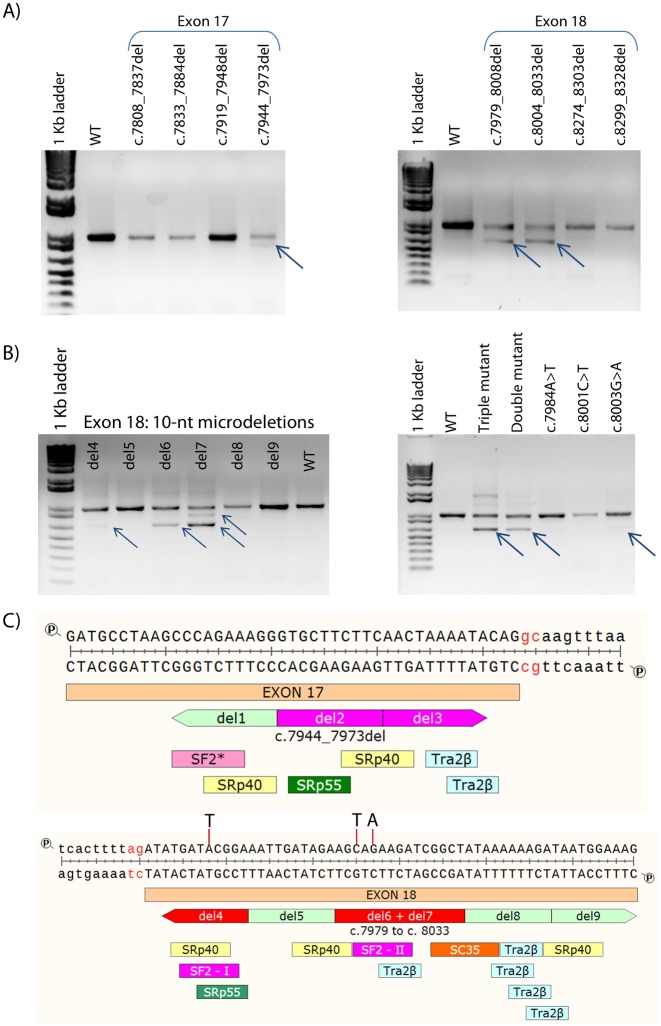
Map of splicing enhancers for recognition of exons 17 and 18. A) Functional mapping of ESEs of exons 17 (left) and 18 (right) by exonic microdeletions (30-nt above) of the wild type minigene MGBR2_ex14-20. cDNA was amplified with primers of *BRCA2* exon 16 (RTBR2_ex16-FW) and vector exon V2 (RTpSAD-RV; amplicon size = 1012 nt) and run in 1.5% agarose gel. Arrows indicate abnormal transcripts. B) Functional analysis of 10-nt microdeletions of exon 18 (left) and single nucleotide substitutions (right) c.7984A>T that disrupts SF2-I, and c.8001C>T and 8003G>A that target SF-II motif (see ESE map below). The triple mutant is the combination c.[7984A>T;8001C>T;8003G>A], and the double one is c.[8001C>T;8003G>A]. C) HSF predictions of putative ESE motifs of exon 17 (above) between cDNA positions 7944 and 7973 and exon 18 (below) between cDNA positions 7979–8008 and 8004–8033. Intronic sequences are in lower case. SF2* motif of exon 17 is detected by the specific SF2/ASF (IgM-*BRCA1*) algorithm of ESEfinder. Red and pink microdeletions alter splicing. Artificial SF2- single-nucleotide substitutions are indicated above exon 18 sequence.

The efficient inclusion of the exons in the mature mRNA may require the presence of enhancer sequences and the binding to SR-proteins [29]. The highest density of active ESEs is near splice sites (~50 nt at both exon ends) with a maximum between 10 and 20 nucleotides from the canonical 5’ and 3’ splice sites of each exon [30]; so DNA variants at these regions have a higher likelihood of disrupting ESEs. Furthermore, previous studies suggested a specific regulation of the donor GC-sites by ESEs [27]. We therefore proceeded to map regulatory sequences involved in exons 17 and 18 processing by functional tests of four exonic 30-nt deletions of each exon. These deletions covered the 5’ and 3’ 55 nucleotides of each exon excluding the first two and last three nucleotides. Three 30-nt microdeletions, c.7944_7973del (exon 17), c.7979_8008del (exon 18) and c.8004_8033del (exon 18), had impacts on splicing (exon 17 or 18 skipping) (Fig 2A), indicating that these sequences probably contain regulatory motifs guiding exon recognition. According to the ESEfinder algorithm [31], these sequences contain several putative enhancer sequences (see Fig 2C), but only SF2/ASF and SRp40 motifs were present in the three deletions suggesting that these SR proteins might be required for competent exon identification. We then carried out the fine mapping of ESEs with additional internal 10-nt deletions of exons 17 [c.7944_7953del (del1), c.7954_7963del (del2) and c.7964_7973del (del3)] and 18 [c.7979_7988del (del4), c7989_7998del (del5), c.7999_8008del (del6), c.8004_8013del (del7; 5-nt overlap between del6 and del7), c.8014_8023del (del8) and c.8024_8033del (del9)] (S2 Table; Fig 2B). The del2 and del3 deletions of exon 17 only disrupted splicing weakly (2.2% and 4.6% of aberrant transcripts, respectively; S3 Table) whereas del1 did not at all. Exon 18 skipping was found at del4, del6 and del7. These segments must therefore contain splicing enhancer sequences. Deletions 5, 8 and 9 of exon 18 did not affect splicing and produced the expected transcripts.

According to the bioinformatics predictions of ESEs (Fig 2C), the common feature of the three positive exon 18 deletions was the presence of two putative ESEs for SF2/ASF (one in the overlapping segment of del6-7) at nucleotides c.7981_7987 (GATACGG) and c.8001_c.8007 (CAGAAGA). We then proceeded to disrupt both motifs by mutagenesis with the following sequence variations: c.7984A>T, c.8001C>T and c.8003G>A, which target conserved nucleotides of the two SF2 motifs (Fig 2C, S2 Table) [31]. We carried out the assays with the triple mutant, a double mutant c.[8001C>T;8003G>A], which targeted the SF2-II site, and the three independent variants (Fig 2B and 2C). Intriguingly, only the triple and the double mutants remarkably impaired splicing with 59.7% and 44.2% of aberrant transcripts (Fig 2B), respectively, but these effects were not observed either with c.7984A>T or c.8001C>T, which did not apparently affect splicing, whereas c.8003G>A only induced weak exon 18 skipping (8.8%), suggesting a synergistic effect of these variants on splicing. We can conclude that these sequences are required for exon 18 recognition so that any variation in these nucleotides may affect splicing and confer breast cancer risk. In order to investigate the participation of SF2/ASF in this process, we initially performed inhibition experiments with siRNAs of splicing factors SF2/ASF, SC35 and Tra2β. Preliminary data unexpectedly suggested a role for SC35 in exons 17 and 18 definitions as well as small contributions of Tra2β and SF2/ASF (S2 Fig). In fact, a putative SC35 motif (GGCTATAA, c.8010-8017) was located at the spliceogenic del7 deletion (Fig 2C).

We also searched for ESE sequences within intron 17. Firstly, we selected a region of 115 nucleotides (c.7976+231_7977-141del) where Human Splicing Finder (HSF) had predicted the presence of a notable concentration of high-scored Tra2β sites (S2 Table), being Tra2β one of the SR-proteins involved in GC recognition in the *BTK* gene [27]. The presence of SREs was also checked in the rest of the intron 17 with another three deletions, c.7976+21_7976+140del, c.7976+136_7976+240del and c.7977-150_7977-21del. However, none of them had an impact on splicing suggesting that regulatory elements of the GC site are not located within intron 17.

### Identification of spliceogenic variants

A total of 221 reported DNA variants (BRCA Share, BIC and 1000 Genomes databases, last accessed date: April 2016) were analyzed with NNSPLICE and HSF. We selected fifty-four out of them (24.4%) on basis of these criteria: splice site disruption or modification, creation of alternative splice sites, disruption of an ESE within positive 30-nt microdeletions, creation of silencers (specifically hnRNPA1 sites) (S2 Table). Remarkably, 36 variants had previously been classified as VUS by the BRCA Share and BIC databases. All the selected DNA changes were introduced into the wild type MGBR2_ex14-20 construct by site-directed mutagenesis except for two (c.7829dup—exon17- and c.8169_8172dup—exon18-) that were disregarded because of the recurrent failure of the mutagenesis experiments.

**Fig 3:**
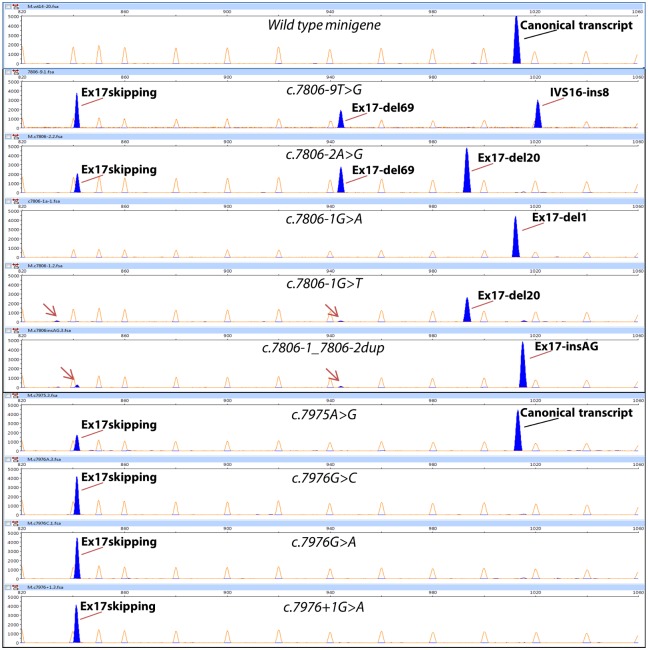
Analysis of transcripts induced by DNA variants from exon 17. cDNA was amplified with primers RTBR2_ex16-FW (*BRCA2* exon 16) (blue peaks) and FAM-labelled RTpSAD-RV (vector exon V2) and electrophoresed on a DNA sequencer with Genescan LIZ 1200 as size standard (orange/faint peaks). Arrows indicate minor aberrant transcripts. Screenshots of electropherograms visualized with the Peak Scanner software v1.0 are shown. Fragment sizes (bp) and relative fluorescent units are indicated on the x- and y-axes, respectively.

**Fig 4:**
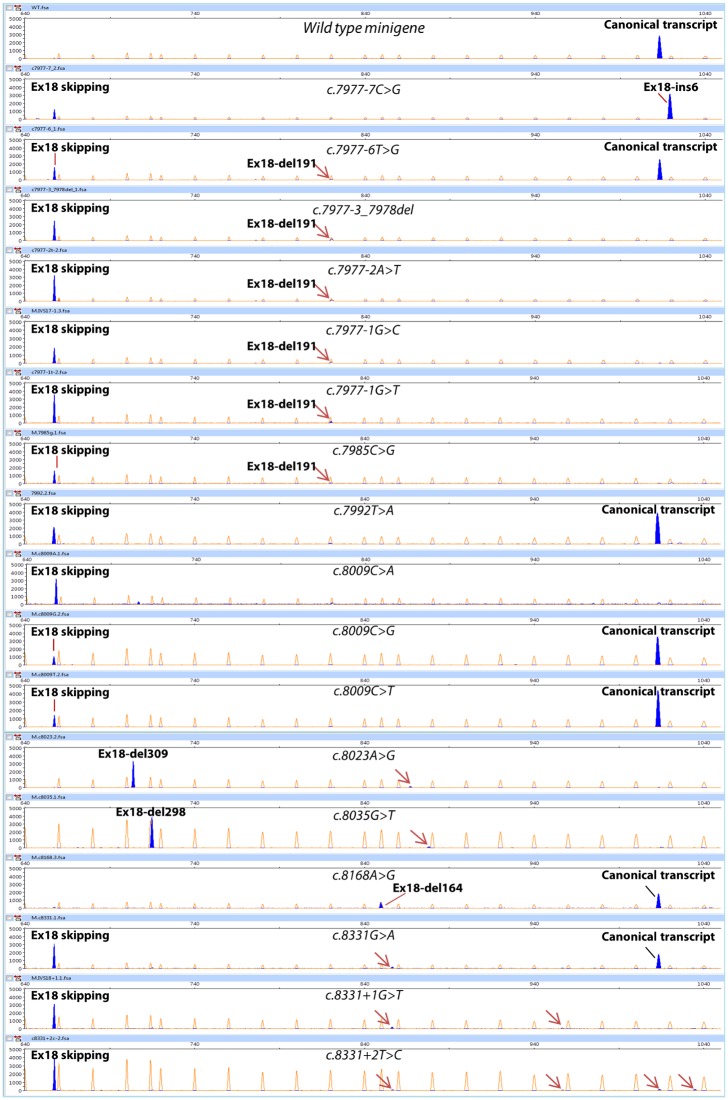
Analysis of transcripts induced by DNA variants from exon 18. cDNA was amplified with primers RTBR2_ex16-FW (*BRCA2* exon 16) (blue peaks) and FAM-labelled RTpSAD-RV (exon V2 of minigene) and electrophoresed on a DNA sequencer with Genescan LIZ 1200 as size standard (orange/faint peaks). Arrows indicate minor aberrant transcripts. Screenshots of electropherograms visualized with the Peak Scanner software v1.0 are shown. Fragment sizes (bp) and relative fluorescent units are indicated on the x- and y-axes, respectively. Electropherograms of c.8007A>G, c.8072C>T and c.8249_8250del are not represented since they show similar patterns (partial exon 18 skipping) to other DNA variants (e.g. c.8009C>G).

Fifty-two variants (17 of exon 17 and 35 of exon 18) were functionally tested in the splicing reporter minigene MGBR2_ex14-20. All the transcripts were quantified to evaluate their possible implication in disease pathogenesis (S3 Table). Only variants with ≥5% of anomalous transcripts were considered as positive. Thirty DNA variants (57.7%) impaired splicing (Table 1, S3 Table, Figs 3 and 4, S3 Fig), whilst another three variants (c.7875A>G, c.7985C>T and c.8042C>G) had weak impacts on splicing (4.7%, 3.3% and 2.3%, respectively; S3 Table). All the splicing outcomes were highly reproducible with low intra-variability (standard deviations <1.8% for 27 variants; S3 Table). Spliceogenic variants consisted of 14 intronic and 16 exonic variants that had previously been predicted as 11 missense, 1 nonsense, 1 frameshift and 3 synonymous variants, confirming that any type of genetic variant can potentially disrupt pre-mRNA processing.

**Table 1:** Splicing outcomes of *BRCA2* exons 17 and 18 variants.

DNA variant ^1^	Motif ^2^	Splicing outcome^3^	RNA effect^4^	Protein Effect^4^
**c.7806-9T>G**	Pyr	Ex17 skipping (41.5%); Ivs16-ins8 (36.3%); Ex17-del69 (22.2%)	r.[7806_7976del,7805_7806ins7806-8_7806–1,7806_7874del]	p. [A2603_R2659del; R2602Sfs*49; A2603_R2625del]
**c.7806-2A>G**	[-] 3’SS	Ex17-del20 (51.8%); Ex17-del69 (28.1%); Ex17 skipping (20.1%)	r.[7806_7825del,7806_7874del,7806_7976del]	p.[A2603Cfs*8;A2603_R2625del;A2603_R2659del]
**c.7806-1G>A**	[-] 3’SS	Ex17-del1 (100%)	r.[7806_7807del]	p.A2603Lfs*45
**c.7806-1G>T**	[-] 3’SS	Ex17-del20 (100%)	r.7806_7825del	p.A2603Cfs*8
**c.7806-1_7806-2dup**	[+]3’SS	Ex17-insAG (92.6%); Ex17 skipping (5.1%); Ex17-del69 (2.3%)	r.[7805_7806insAG,7806_7976del,7806_7874del]	p. [A2603Gfs*46; A2603_R2659del; A2603_R2625del]
c.7975A>G	[-] 5’SS	**CT** (73.8%); Ex17 skipping (26.2%)	r.[7975a>g,7806_7976del]	p. [R2659G;A2603_R2659del]
**c.7976G>C**	[-] 5’SS	Ex17 skipping (100%)	r.7806_7976del	p.A2603_R2659del
**c.7976G>A**	[-] 5’SS	Ex17 skipping (100%)	r.7806_7976del	p.A2603_R2659del
**c.7976+1G>A**	[-] 5’SS	Ex17 skipping (100%)	r.7806_7976del	p.A2603_R2659del
**c.7977-7C>G**	[+]3’SS/Pyr	Ex18-ins6 (78.4%); exon 18 skipping (21.6%)	r.[7976_7977ins6,7977_8331del]	p.[Y2658_R2659insSF; Y2660Ffs*43]
c.7977-6T>G	Pyr	**CT** (66.7%); Ex18 skipping (31%); ex18-del191 (2.3%)	r. [=, 7977_8331del,7977_8167del]	p. [=; Y2660Ffs*43;Y2660Wfs*6]
**c.7977-3_7978del**	[-] 3’SS	Ex18 skipping (90%) ex18-del191 (10%)	r.[7977_8331del,7977_8167del]	p.[Y2660Ffs*43;Y2660Wfs*6]
**c.7977-2A>T**	[-] 3’SS	Ex18 skipping (93.3%); ex18-del191 (6.7%)	r.[7977_8331del,7977_8167del]	p.[Y2660Ffs*43;Y2660Wfs*6]
**c.7977-1G>T**	[-] 3’SS	Ex18 skipping (91.5%); ex18-del191 (7%); ex18-del236 (1.5%)	r.[7977_8331del,7977_8167del,7977_8212del]	p.[Y2660Ffs*43;Y2660Wfs*6;R2659Sfs*26]
**c.7977-1G>C**	[-] 3’SS	Ex18 skipping (89.8%); Ex18-del191(10.2%)	r.[7977_8331del,7977_8167del,?]	p.[Y2660Ffs*43; Y2660Wfs*6;?]
**c.7985C>G**	[-]ESE/[+]ESS	Ex18 skipping (90.2%); Ex18-del191 (5%); others (4.8%)	r.[7977_8331del,7977_8167del,?]	p.[Y2660Ffs*43; Y2660Wfs*6;?]
c.7988A>T	[+]5’SS [-]ESE	**CT** (84.2%); Ex18 skipping (8.6%) + others (7.2%)	r.[7988a>u,7977_8331del,?]	p.[E2663V;Y2660Ffs*43;?]
c.7992T>A	[-]ESE/[+]ESS	**CT** (68.6%); ex18 skipping (31.4%)	r.[7992u>a,7977_8331del]	p. [=; Y2660Ffs*43]
c.8007A>G	[-]ESE/[+]ESS	**CT** (84.8%); ex18 skipping (15.2%)	r.[8007a>g,7977_8331del]	p. [=; Y2660Ffs*43]
**c.8009C>A**	[-]ESE/[+]ESS	Ex18 skipping (91.2%); Ex18-del191(4.8%); **CT** (4%)	r.[7977_8331del,7977_8167del,8009c>a,?]	p. [Y2660Ffs*43; Y2660Wfs*6;S2670*;?]
c.8009C>T	[-]ESE/[+]ESS	**CT** (76.6%); ex18 skipping (23.4%)	r.[8009c>u,7977_8331del]	p.[S2670L;Y2660Ffs*43]
c.8009C>G	[-]ESE/[+]ESS	**CT** (79.9%); ex18 skipping (20.1%)	r.[8009c>g,7977_8331del]	p.[S2670W;Y2660Ffs*43]
**c.8023A>G**	[+]5’SS	Ex18-del309 (93%); other aberrant transcripts (7%)	r.[8023_8331del,?]	p.[Ile2675_K2777del;?]
**c.8035G>T**	[+]5’SS	Ex18-del298 (93.6%); 878-nt transcript (4%); **CT**: 2.4%	r.[8034_8331del,?]	p.[D2679Ffs*43;?]
c.8072C>T	[-]ESE/[±]ESS	**CT** (94.9%); ex18 skipping (5.1%)	r.[8072c>u,7977_8331del]	p.[S2691F;Y2660Ffs*43]
c.8168A>G	[+]5’SS	**CT** (69.6%); Ex18-del164 (25.9%) /Ex18 skipping (4.5%)	r.[8168a>g,8168_8331del,7977_8331del]	p.[D2723G;G2724Ffs*3; Y2660Ffs*43]
c.8249_8250del	[-]ESE/[-]ESS	**CT** (93.0%); ex18 skipping (7.0%)	r.[8249_8250del,7977_8331del]	p.[K2750Asnfs*13; Y2660Ffs*43]
**c.8331G>A**	[-] 5’SS	Ex18 skipping (52%); **CT** (40.7%); aberrant transcripts (7.3%)	r.[8331g>a,7977_8331del,?]	p. [=; Y2660Ffs*43;?]
**c.8331+1G>T**	[-] 5’SS	Ex18 skipping (81%); Ex18-del157 (6.4%); ex17-del151+ex18 skipping (6.1%); ivs17 58-nt retention+Ex18 skipping (3.7%);others (2.8%)	r.[7977_8331del,8175_8331del,7826_8331del, 7977_8331delins7976+1_7976+58]	p. [Y2660Ffs*43;W2725*; G2609Dfs*4; Y2660Qfs*3]
**c.8331+2T>C**	[-] 5’SS	Ex18 skipping (87.1%); ex17-del151+ex18 skipping (12.9%)	r.[7977_8331del,7826_8331del]	p.[Y2660Ffs*43; G2609Dfs*4]

^1^ Bold type variants indicate proposed causal or likely causal variants attending to the guidelines of the American College of Medical Genetics and Genomics (see also S4 Table);

^2^ Affected motifs: 3’SS, 3’ splice site; 5’SS: 5’ splice site; Pyr, polypyrimidine tract; ESE, Exonic Splicing Enhancer; ESS, Exonic Splicing Silencer; [-] disruption; [+] creation.

^3^ The proportion of each transcript is indicated between parentheses; CT: Canonical transcript.

^4^ HGVS nomenclature.

According to the prediction software and their location,15 variants disrupted the canonical 3’ and 5’ splice sites (c.7806-2A>G, c.7806-1G>A, c.7806-1G>T, c.7806-1_7806-2dup—previously reported as c.7806insAG-, c.7975A>G, c.7976G>C, c.7976G>A, c.7976+1G>A, c.7977-3_7978del, c.7977-2A>T, c.7977-1G>T, c.7977-1G>C, c.8331G>A, c.8331+1G>T and c.8331+2T>C), three disrupted the polypyrimidine tract (c.7806-9T>G, c.7977-7C>G and c.7977-6T>G), three created novel active splice sites (c.8023A>G, c.8035G>T and c.8168A>G, but also c.7977-7C>G and c.7806-1_7806-2dup—see above-), seven affected enhancer or silencer motifs (c.7992T>A, c.8007A>G, c.8009C>A, c.8009C>T, c.8009C>G, c.8072C>T and c.8249_8250del, all of them in exon 18) and two were presumed to alter ESE/ESS motifs and generate alternative sites (c.7985C>G—weak 3’ss- and c.7988A>T—strong 5’ss-) that actually were not used, so they should be considered as ESE/ESS-variants. Seven of the ESE/ESS variants were placed into the positive ESE-containing microdeletions c.7979_8008del30 and c.8004_8033del30, spanning a 25-nt interval of exon 18 (c.7985-8009).

### Characterization of aberrant transcripts

**Fig 5:**
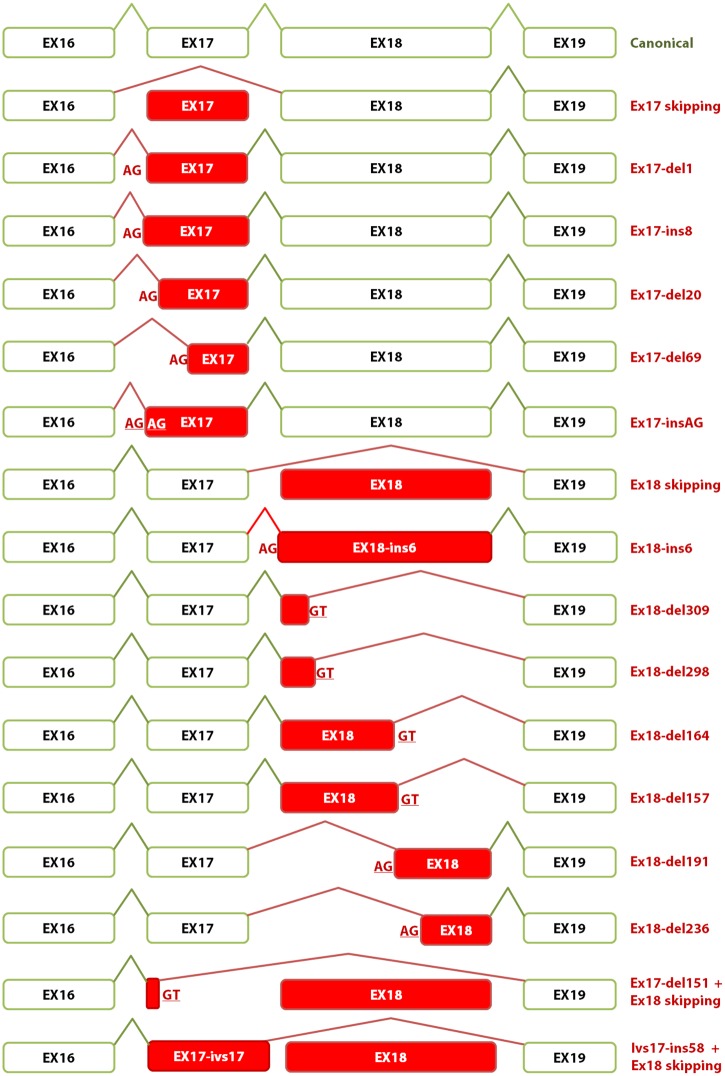
Schematic representation of splicing events and transcripts generated by wild type and mutant minigenes. Exons and splicing reactions exons are represented by boxes and broken lines, respectively. Anomalous/skipped exons and aberrant events are shown in red.

Variants of exon 17 and flanking intronic sequences rendered 6 different abnormal transcripts (Fig 5): ex17 skipping, ex17-ins8 (alternative intronic acceptor 8 nt upstream), ex17-del1 (novel acceptor 1 nt downstream), ex17-del20 (alternative acceptor 20 nt downstream), ex17-del69 (alternative acceptor 69 nt downstream) and ex17-insAG (novel acceptor 2 nt upstream), where exon 17 skipping was the most abundant event. Mutations at exon 18 and contiguous sequences induced more than 10 different aberrant transcripts (Fig 5): ex18 skipping, ex18-ins6 (novel intronic acceptor 6 nt upstream), ex18-del191 (alternative acceptor 191 nt downstream), ex18-del309 (new donor 309 nt upstream), ex18-del298 (new donor 298 nt upstream), ex18-del164 (new donor 164 nt upstream), ex18-del157 (use of cryptic donor 157 nt upstream), and rare phenomena such as ex17-del20+ex18 skipping (cryptic acceptor plus skipping), ivs17_58 nt retention+ex18 skipping (intronic cryptic donor plus skipping), one 878-nt transcript as well as other uncharacterized aberrant transcripts. Exon 18 skipping was the most frequent outcome (19 out of 21 variants induced it). Twelve transcripts would introduce premature termination codons (PTC).

## Discussion

The functional and clinical classifications of DNA variants of breast cancer genes provide essential information for clinical management of patients and asymptomatic carriers. The identification of VUS in patients hampers the genetic counseling of BC families since the result of the genetic test is ambiguous. Pathogenicity of genetic alterations is usually assumed for sequence variants that introduce PTCs, such as frameshift and nonsense mutations, or some missense changes that disrupt protein function. However, many other DNA variants can affect disease risk as other mechanisms of gene expression, such as pre-mRNA processing, are affected. In fact, many germ-line variants of human disease genes have already been associated with aberrantly spliced mRNAs that encode defective proteins [32].

In this work, we have carried out one of the most comprehensive studies of the correlation between aberrant splicing and breast cancer, whereby we evaluated the impact on splicing of 52 DNA variants of *BRCA2* exons 17 and 18 with the stable seven-exon minigene MGBR2_ex14-20. We have shown that spliceogenic variants are relatively abundant in exons 17 and 18 (30/52 tests), representing ~15% of all reported variants of these exons and flanking intronic sequences. This rate triplicates the frequency of splice sites variants (<10 bp from exon) at the BRCA Share database (131/2818 recorded variants, 4.6%). Remarkably, these 30 variants were found in 157 different records of the BIC and BRCA Share databases. A high prevalence of spliceogenic variants has been reported in several disease genes, such as *NF1*, where up to 44% of patients carry such type of alterations [23,33]. So far we have performed lymphocyte or minigene RNA assays of 188 variants, 93 of which (49.5%) disrupted splicing [19,24,25].

We have once more demonstrated the high capacity, robustness and simplicity of splicing reporter minigenes, with the following advantages: a) observation of a single-mutant allele effect without the interference of the wild type counterpart in patient samples; b) precise quantification of all anomalous transcripts by inhibiting the NMD; c) high capacity of this simple technical approach (Cloning-Mutagenesis-Functional Assay) [19,24,25,28,34]; d) one single minigene-construct allows the analysis of multiple variants from different exons (seven exons in this case); e) high reproducibility of physiological/pathological splicing patterns by virtue of keeping the genomic context of each exon. This was supported by comparing our results with previous studies based on patient RNA data. We found at previous reports that 8 DNA variants under study yielded, all of them, the same or similar results for patient RNA and minigene assay. Thus, DNA variants: c.7806-9T>G (exon 17 skipping+ex17-del69+ivs16-ins8) [35], c.7975A>G (partial exon 17 skipping) [36], c.7976G>A and c.7976G>C (total exon 17 skipping) [37,38], c.7988A>T (partial exon 18 skipping) [38], c.7992A>T (partial exon 18 skipping) [39], c.8023A>G (ex18-del309) [21] and c.8168A>G (partial ex18-del163) [40] displayed the same splicing patterns in patient RNA and minigene MGBR2_ex14-20, lending further support to the reproducibility of minigene results. Furthermore, a recent splicing study of 30 variants of *MLH1*, *MSH2*, *MSH6*, and *PMS2* genes (Lynch syndrome) showed that outcomes of patient RNA and minigene assays were almost identical [41]. Thus, we can conclude that the minigene strategy is sensitive and specific, so its use is suitable for the initial characterization of the splicing anomalies [21]. Nevertheless, it should be considered only as a supportive test rather than a confirmatory one since minigene results should be confirmed in patient RNA or even at protein levels whenever possible. However, it is also worth mentioning that splicing outcomes from patient RNA may be biased by technical limitations, physiological alternative transcripts, and cell-type specific differences between the patient sample (principally leukocyte RNA) and the affected tissue [42].

Finally, we have already cloned 43 out of the 50 exons of *BRCA1/2* in the following 6 constructs: *BRCA1* exons 2 to 10, and 12 to 23, *BRCA2* exons 2 to 9, 9 to 10 (http://www.ibgm.med.uva.es/servicios/servicio-de-splicing-minigenes/),14 to 20 (this study) and 19 to 27 [24]. Any variant of such exons can be easily tested with a straightforward protocol in less than two weeks.

### Types of variants and splicing regulatory elements

Splicing is specifically regulated by a dense array of motifs and splicing factors so that an important message of our study is that any nucleotide change has the potential of disrupting this process. In fact, the 30 positive changes comprised 14 intronic and 16 exonic variants including 11 missense, 1 frameshift, 1 nonsense and 3 synonymous predicted changes [43]. Synonymous variations are particularly interesting since they have traditionally been considered as neutral. Many sequence variations affect disease risk, including synonymous variants that, actually, may have unexpected deleterious effects over the splicing and protein translation mechanisms [44,45]. It has been shown that these variants account for 6–8% of all driver mutations in oncogenes, where about half of them impair splicing [46]. Conversely, protein truncating variants (nonsense) are directly classified as deleterious though we have herein shown that the associated-nucleotide changes can affect splicing regulatory elements, so we could observe a “dangerous” unclassifiable splicing effect whenever they induced in-frame deletions of an exon.

With regard to the affected splicing motifs of positive variants (Table 1, S2 Table) and taking into account the bioinformatics and splicing outcomes, we can conclude that 15 variants affected the natural 5’ and 3’ splice sites, three the polypyrimidine tract, three created novel alternative splice sites and 9 affected ESE/ESS motifs. The NNSplice, MaxEnt and HSF algorithms accurately anticipated the splice site disruptions and the generation of novel active sites, but the splicing outcomes were absolutely unpredictable reinforcing the current need of functional assays. The characterization of the physiological alternative splicing events of *BRCA1* and *BRCA2* [14,47] and improved computer tools will help to estimate the aberrant transcripts that a particular DNA variant may generate. It is also worthy to mention that three variants of the polypyrimidine tract, c.7806-9T>G, c.7977-7C>G and c.7977-6T>G, produced defective splicing. Pyrimidine to Purine changes at this element are critical for exon recognition as we had previously described [25]. However, these modifications are barely identified by the splicing software with slight reductions of the splice site score (S2 Table). For example, NNSplice of c.7806-9T>G calculated a weak decrease of the 3’ splice site score of exon 17 from 0.95 to 0.83, yet it was associated with a total splicing disruption.

*In silico* predictions of ESE/ESS motifs, which are constituted by short-degenerate sequences, showed low sensitivity. Nevertheless, there have been recently postulated two *in silico* approaches, ΔtESRseq and ΔHZ_EI_, that accurately detect potential ESE-variants [43]. We have found that 9 out of 28 pre-selected ESE/ESS variants affected splicing, so we have even improved its accuracy with respect to former studies by virtue of the functional mapping by microdeletions that has proven to be an exceptional method to refine ESE-variant selection. This strategy revealed the presence of operating ESEs in intervals c.7944-7973 (exon 17) and c.7979-8008 and c.8004-8033 (exon 18). Remarkably, 7 out of 9 ESE/ESS variants are placed within these intervals of exon 18 confirming the value of preliminary ESE-mapping to choose candidate variants and to fine map regulatory sequences. Interestingly, only the triple (c.7984, c.8001 and c.8003) and double (c.8001 and c.8003) mutants of SF2 sites significantly affected splicing whereas single mutants did not or did only weakly, suggesting a precise and compound control of exon 18 processing, where ESE sequences might act cooperatively. In this regard, while two possible SF2/ASF sites were bioinformatically predicted (c.7981_7987, GATACGG, and c.8001_c.8007, CAGAAGA) (Fig 2C), preliminary siRNAs experiments suggested the participation of the splicing factor SC35 in the regulation of exons 17 and 18 (S2 Fig), which is also involved in the regulation of a pathological GC site of the *BTK* gene [27]. Nevertheless, the definite identification of the splicing factors involved in exons 17 and 18 processing should be carried out by further siRNA and pulldown assays [48]. Independently of the factors involved, these data allowed us to underline three small DNA segments (c.7944-7973, c.7979-7988 and c.7999-8013) where spliceogenic ESE-variants may occur. Given the poor precision of ESE/ESS-prediction software (12.2% of selected variants) [19,24], these data will provide a very valuable information for genetic counselors with a view to selecting specific exonic mutations within those intervals for splicing assays.

Donor-GC sites, such as that of exon 17, have been linked to alternative splicing [26] so that they require the control by factors that promote their efficient selection [27]. Certainly, exons 17 and 18 undergo naturally-occurring alternative splicing producing minor transcripts Δ18 and Δ17+18 [28], although in our study Δ18 was only detected at even lower levels in the wild type minigene (<1%; S3 Table), together with the full-length transcript (≥99%). This may probably be due to: i) the genomic context that influences exon recognition [15]; ii) tissue-dependent alternative splicing as we used different host cells (MCF-7 vs. HeLa); and iii) RNA preparation and storage conditions, primer design, PCR conditions, and PCR product detection methodology can introduce small variations in splicing isoform ratios as previously reported [14,42].

### Clinical interpretation of DNA variants

Identification of pathogenic variants with impact on splicing will aid in breast cancer prediction, prevention and surveillance, but the clinical interpretation of the splicing outcomes of candidate variants is a particularly complex task. It is accepted that a variant would be considered likely pathogenic when it causes a majority of aberrant RNA isoforms and generates a stop codon or loss of a known functional domain. The identification of numerous anomalous transcripts of exons 17 and 18 and the production of ≥2 transcripts by many variants are proofs of this arduous undertaking.

Twelve transcripts introduced a frameshift in the open reading frame and a PTC so they inactivated *BRCA2*: ex17-del1, ex17del20, ex17ins8, ex17insAG, ex18 skipping, ex18-del191, ex18-del298, ex18-del164, ex18-del157, ex18-del236, ex17-del151+ex18 skipping and ivs17_58-nt retention+ex18 skipping. Conversely, exon 17 skipping, ex17-del69, ex18-ins6 and ex18-del309 kept the reading frame with *a priori* unknown impact on *BRCA2* function. Exon 17 skipping and ex17-del69 led to deletions of 57 and 23 amino acids, respectively, at the essential α-helical domain of the *BRCA2* protein (amino acids 2479 to 2667). This domain facilitates *BRCA2* binding to single-stranded and double-stranded DNA [49]. Moreover, 30 out of the 57 residues encoded by exon 17 are strictly conserved from sea urchin to human revealing its importance for *BRCA2* activity (IARC *BRCA2* alignment; http://agvgd.iarc.fr/BRCA2_Align.htm). Likewise, it has been shown that the loss of exon 17 inactivates *BRCA2* function [37]. Furthermore, exon 17 variant c.7976G>A, which is associated with total exon 17 skipping, reached odds of causality of >3,000:1 [38]. Consequently, we can infer that the rest of the variants with ex17 skipping as the unique transcript, such as c.7976G>C and c.7976+1G>A, are also likely pathogenic. Moreover, variants c.7806-9T>G and c.7806-2A>G with at least 3 abnormal transcripts, including ex17 skipping, could also be considered as likely pathogenic, given that the other transcripts disrupt the reading frame (Ivs16ins8 or ex17-del20) or leads to in-frame loss of 23 aminoacids (ex17-del69), 13 of which are strictly conserved. The RNA isoform ex18-ins6 would insert new amino acids Ser-Phe between Tyr2658 and Arg2659. Precisely, amino acids from Val2652 to Asp2661 are conserved from sea urchin, and two missense changes at this protein segment, p.Leu2653Pro and p.Arg2659Lys were formerly classified as deleterious [38,50]. Consequently, transcript ex18-ins6 might have a deleterious impact on *BRCA2* function but it is required further protein function studies. Finally, abnormal transcript ex18-del309 was predicted to cause an in-frame deletion of 103 amino acids between codons Ile2675 and Lys2777 of the OB1 (oligonucleotide ssDNA-binding fold) motif at the DNA binding domain of *BRCA2*, 24 of which are strictly conserved from sea urchin. Variant c.8023A>G, which induced ex18del309, had previously been classified as pathogenic [21] (BIC and UMD databases), so this transcript disrupts *BRCA2* function. Also, c.8331G>A might be an important risk allele as abnormal transcripts almost reach 60%, which is the suggested threshold for severe splicing aberrations [51]. According to the guidelines of the American College of Medical Genetics and Genomics (ACMG), [52] 12 spliceogenic variants were classified as pathogenic (c.7806-2A>G, c.7806-1G>A, c.7806-1G>T, c.7806-1_7806-2dup, c.7976+1G>A, c.7977-3_7978del, c.7977-2A>T, c.7977-1G>T, c.7977-1G>C, c.8009C>A, c.8331+1G>T and c.8331+2T>C) and 8 as likely pathogenic (c.7806-9T>G, c.7976G>C, c.7976G>A, c.7977-7C>G, c.7985C>G, c.8023A>G, c.8035G>T and c.8331G>A), under the splicing viewpoint (S4 Table). Remarkably, all of them account for 72 independent records at the mutation databases (S4 Table) and nine of them had been classified as VUS. Reclassification of VUS as deleterious will notably increase the number of HBOC families who may benefit from tailored preventive and prophylactic measures as well as new targeted therapies, such as Poly (ADP-ribose) polymerase (PARP)-inhibitors, for patients with *BRCA1*/2 associated cancers [53].It is also worthy to mention that causal and likely causal splicing variants account for a remarkable 30.8% (20/65) of all predicted pathological variants of exons 17 and 18 at the *BRCA2* Share database (S5 Table), representing the second more frequent type of causal variants after frameshift mutations (44.6%).

On the other hand, two variants with weaker splicing alterations, c.8168A>G/ p.Asp2723Gly (30%) and c.8249_8250del (7%), were previously classified as likely pathogenic (protein function and truncation, respectively) [38], so their pathogenicity may probably be due to a double mechanism: protein inactivation and splicing disruption, like *BRCA1* c.5123C>A (p.A1708E) [25,54]. Likewise, c.8009C>A was previously classified as causal because of its predicted nonsense change (p.Ser2670X), but it actually induces 96% of aberrant transcripts so it should be reclassified as a spliceogenic variant. Reclassification of missense and protein truncation variants as splicing alterations might also have an effect in their penetrance and expressivity. Taken together, 8 spliceogenic variants remain classified as VUS since relevant proportions of the full-length transcript were detected (c.7975A>G, c.7977-6T>G, c.7988A>T, c.7992T>A, c.8007A>G, c.8009C>T, c.8009C>G and c.8072C>T) (Table 1; S3 and S4 Tables). It is complex to interpret the role of variants with partial splicing anomalies in HBOC under the clinical perspective as they will require more studies to elucidate it. Nevertheless, we can speculate that they represent low BC risk alleles that might interact with other susceptibility and protector alleles to modify the overall BC risk. The incorporation of all these data into a single integrated model of BC risk would improve disease prediction and prevention.

In conclusion, dysregulation of splicing should be considered as a primary mechanism of gene inactivation to be investigated in human disease genes. Spliceogenic variants are comparatively abundant in *BRCA2* exons 17 and 18 because recognition of both exons additionally requires the regulation of specific ESE motifs in exons 17 and 18, whose abolitions drive splicing aberrations. Furthermore, the pSAD-based minigenes are useful tools for molecular diagnostics and genetic counseling of hereditary breast/ovarian cancer or other genetic disorders as well as for the basic research on the splicing process. Hence, RNA assays supply essential information for the clinical interpretation of variants that should be incorporated in the genetic counselling of human hereditary diseases.

## Materials and methods

### Databases and bioinformatics analyses

*BRCA2* variants of breast/ovarian cancer patients were available from the BIC (https://research.nhgri.nih.gov/projects/bic/Member/index.shtml) and the BRCA Share databases (last accessed date 2016/04/01; http://www.umd.be/BRCA2/) [55]. Variants of intron 17 were collected from the 1000 Genomes database (http://browser.1000genomes.org/Homo_sapiens/Gene/Summary?db=core;g=ENSG00000139618;r=13:32889611-32973805;t=ENST00000380152). Variant descriptions were according to the *BRCA2* GenBank sequence NM000059.1 and the guidelines of the Human Genome Variation Society (HGVS; http://www.hgvs.org/mutnomen/).

Mutant and wild type (wt) sequences were analyzed with NNSPLICE (http://www.fruitfly.org/seq_tools/splice.html) [56], and Human Splicing Finder version 3.0 (HSF; http://www.umd.be/HSF3/)[57], which includes algorithms for splice sites, silencers and enhancers [31,58–62].

### Minigene construction

MGBR2_ex14-20 was assembled in four steps by overlapping extension PCR or classical restriction digestion/ligation cloning with three intermediate constructs: MGBR2EX17-18, MGBR2EX16-18, and MGBR2EX16-20. All the inserts were amplified with Phusion High Fidelity polymerase (Thermo Fisher Scientific, Waltham, MA, USA) and primers indicated on S1 Table. Exons 17–18 were subcloned into the pSAD vector by overlapping extension PCR. Then, exon 16 was added by the same technique. Exons 19–20 were inserted between the Xhol and BamHI restriction sites of the 16–18 construct. Finally, exons 14–15 were introduced using the EagI and SacI restriction sites. All clones were functionally checked in MCF-7 cells.

### Site-directed mutagenesis

DNA variants were introduced with the QuikChange Lightning kit (Agilent, Santa Clara, CA). The wt minigene MGBR2_ex14-20 was used as template to generate 52 BIC/BRCA Share DNA variants as well as seventeen exonic (17 and 18) and four intronic (ivs17) microdeletions (S2 Table). The first two and the last three nucleotides of each exon were always preserved to avoid any disruptions of the canonical acceptor and donor sites, respectively. Deletions were introduced by PCR-mutagenesis with chimeric 50-60mer primers containing 25–30 nucleotides of each end of the deletion.

### Transfection of eukaryotic cells

Approximately 2x10^5^ MCF7 cells were grown to 90% confluency in 0.5 mL of medium (MEME, 10% Fetal Bovine Serum, 2 mM glutamine, 1% Non-essential amino acids and 1% Penicillin/Streptomycin) in 4-well plates (Nunc, Roskilde, Denmark). Cells were transiently transfected with 1 μg of each minigene and 2 μL of Lipofectamine 2000 or low toxicity Lipofectamine (Life Technologies, Carlsbad, CA). To inhibit nonsense mediated decay (NMD), cells were incubated with cycloheximide (Sigma-Aldrich, St. Louis, MO) 300 μg/mL for 4 hours. RNA was purified with the Genematrix Universal RNA Purification Kit (EURx, Gdansk, Poland) with on-column DNAse I digestion to degrade genomic DNA that could interfere in RT-PCR.

### RT-PCR of minigenes

Retrotranscription was carried out with 400 ng of RNA and RevertAid H Minus First Strand cDNA Synthesis Kit (Life Technologies), using gene specific primer RTPSPL3-RV (5’TGAGGAGTGAATTGGTCGAA 3’). Samples were incubated at 42°C for 1 hour, and reactions were inactivated at 70°C for 5 min. Then, 1–2 μl of the resultant cDNA were amplified with SD6-PSPL3_RTFW (5’-TCACCTGGACAACCTCAAAG-3’) or RTBR2_ex16FW (5’-TATGGACTGGAAAAGGAATAC-3’) and RTpSAD-RV (Patent P201231427, CSIC) (sizes: 1012 and 1806 bp, respectively) using Platinum Taq DNA polymerase (Life Technologies). Samples were denatured at 94°C for 2 min, followed by 35 cycles consisting of 94°C for 30 sec, 59°C for 30 sec, and 72°C (1 min/kb), and a final extension step at 72°C for 5 min. Sequencing reactions were performed either using the kit BigDye Terminator v3.1 Cycle Sequencing Kit (Applied Biosystems) following the manufacturer's instructions or by the sequencing facility of Macrogen Europe (Amsterdam, The Netherlands). All transcripts from exon 18 microdeletion c.8004_8013del were subcloned into the pJet1.2 PCR cloning vector (Thermo Fisher Scientific) and sequenced.

In order to quantify all transcripts, semi-quantitative fluorescent PCRs were undertaken at least in triplicate (>234 assays were performed of 52 natural plus 26 artificial mutants; S2 Table) with primers RTBR2_ex16-FW and FAM-RTpSAD-RV and Platinum Taq DNA polymerase (Life Technologies) under standard conditions except that 26 cycles were herein applied [19,63]. FAM-labeled products were run with LIZ-1200 Size Standard at the Macrogen facility and analyzed with the Peak Scanner software V1.0. Only peaks with heights ≥ 50 RFU (Relative Fluorescence Units) were taken into account. Peak areas were used to quantify the relative abundance of each transcript that was the average of at least three experiments [19].

## Supporting information

S1 Table Primers used for the construction of the MGBR2_ex14-20 minigene. (PDF)

S2 Table Bioinformatics analysis and mutagenesis primers of selected reported and artificial DNA variants of exons 17 and 18. (DOCX)

S3 Table Relative quantification of transcripts induced by DNA variants and microdeletions of *BRCA2* exons 17and 18 in MCF-7 cells. (PDF)

S4 Table Analysis of the 52 assayed variants according to the guidelines of the American College of Medical Genetics and Genomics (ACMG). (PDF)

S5 Table Distribution of pathogenic/likely pathogenic variants by type according to the BRCA Share database and this study. (PDF)

S1 Fig Capillary electrophoresis of the [FAM]-RT-PCR products generated by the mutant c.7976+2C>T (canonical donor GT site, above) and the wild type (below) minigenes. The blue peaks denote the full-length transcripts induced by both minigenes and the Genescan 1200 Size Standard (Applied Biosystems) is shown as orange peaks.
(TIF)

S2 Fig Impact of RNA interference-mediated depletion of splicing factors SC35, SF2/ASF and Tra2β on splicing of minigene MGBR2_ex14-20. About 1.5x10^5^ MCF7 cells were subjected to a two-hit transfection with Oligofectamine (Thermo Fisher Scientific) and custom-made small interfering RNA (siRNA) at a final concentration of 0.08μM on day 2, the minigene was transfected with Lipofectamine (Thermo Fisher Scientific) on day 4, and RNA was extracted on day 5. The following siRNAs were used: SC35 (aauccaggucgcgaucgaa), SF2/ASF (acgauugccgcaucuacgu), Tra2β (ggaucuucgugaaguauuu) and a control luciferase siRNA. RT-PCR products were run on an agarose gel (1.0%) and stained with Ethidium Bromide. Several aberrant transcripts are visualized, some of which are indicated by arrows.
(TIF)

S3 Fig High resolution of capillary electrophoresis of fluorescent RT-PCRs. Electropherograms of the wild type minigene and mutations c.7806-1G>A and c.7806-1_7806-2dup were overlaid. Transcripts ex17-del1 and ex17-insAG differed in size by only 1 and 2 nucleotides, respectively, from the canonical transcript despite the large size (1,012 nt) of the RT-PCR products. The Liz-1200 size standard is shown as orange peaks.
(TIF)
