# Supplementary material for: Functional classification of DNA variants by hybrid minigenes: Identification of 30 spliceogenic variants of BRCA2 exons 17 and 18
Source: PLoS Genet. 2017 Mar 24;13(3):e1006691. doi: 10.1371/journal.pgen.1006691 (PMC5384790; doi:10.1371/journal.pgen.1006691)
Supplement: S1 Table — (PDF) [file pgen.1006691.s001.pdf]

S1 Table. Primers used for the construction of the MGBR2_ex14-20 minigene.

Primer	5'-3' Sequence	Size
MGBR2_ex14-15-SacIFW	CACACAGAGCTCTGAAGATGTGAAGGTGAGAGAA	2405
MGBR2_ex14-15-EagIRV	CACACACGGCCGGTTCAGGGCTATCAGTTATTCA	
MGBR2_ex17-18_Ins-FW	GCTCTAGAACTAGTGGATCCCCCGGTCAGTATGATACTTTGATACATGT	1556
MGBR2_ex17-18_Ins-RW	ATAAGCTTGATATCGAATTCCTGCAGTGGAATAGGGATCTGATCAA	
MGBR2_ex16_Ins-FW	GCTCTAGAACTAGTGGATCCCCCGGCCCTTTTGTTTCCCATCTAAGT	805
MGBR2_ex16_Ins-RW	GACATGTATCAAAGTATCATACTGAATAAATGCCTAAGAAAAATGT	
MGBR2_ex19-20_XhoI-FW	CACACACTCGAGATAGCATTAAGAACTTGTAGCA	1188
MGBR2_ex19-20-KpnI-RV	CACACAGGTACCATTACAAATGGCTTAGACCTGA	
