# Supplementary material for: Functional classification of DNA variants by hybrid minigenes: Identification of 30 spliceogenic variants of BRCA2 exons 17 and 18
Source: PLoS Genet. 2017 Mar 24;13(3):e1006691. doi: 10.1371/journal.pgen.1006691 (PMC5384790; doi:10.1371/journal.pgen.1006691)
Supplement: S2 Table — (DOCX) [file pgen.1006691.s002.docx]

**S2 Table**. Bioinformatics analysis and mutagenesis primers of selected reported and artificial DNA variants of exons 17 and 18.

| **DNA variant^1^** | **Type** | | **Previous Classification** | **Bioinformatics^2,3^** | | | | **Primers for Site-directed Mutagenesis (5’- 3’)** |
| --- | --- | --- | --- | --- | --- | --- | --- | --- |
|  |  |  |  | **Acceptor/Donor** | **ESEs** | **ESSs** | |  |
| **EXON 17** |  | |  |  |  |  | |  |
| c.7806-40A>G | Intronic | | VUS | HSF: [-]branch site 45 nt upstream (67.63→38); | [+] SRp55 (74.82); [+] PESE | [-] 2 Sironi ESS; [+] Fas-ESS; [-] hnRNPA1 (66.19) | | GTTGAATTCAGTATCATCCTGTGTGGTTTTTATGATAATAT  ATATTATCATAAAAACCACACAGGATGATACTGAATTCAAC |
| c.7806-9T>G | Intronic | | VUS | NNS: ↓3’ ss (0.95→0.83, [+] 3’ss=0.69.  MaxEnt: 3’ss:8.33→1.73; [+] 3’ss=6.01 | - | - | | ATGATAATATTCTACTTTTAGTTGTTCAGGGCTCTGTGTGA  TCACACAGAGCCCTGAACAACTAAAAGTAGAATATTATCAT |
| c.7806-2A>G | Intronic | | Causal | [-] 3’ ss (NNS: 0.95 →<0.4) | [-] 9G8 (59.87) | [+] 3 Sironi ESS;  [-] hnRNPA1(82.86) | | TCTACTTTTATTTGTTCGGGGCTCTGTGTGACACT  AGTGTCACACAGAGCCCCGAACAAATAAAAGTAGA |
| *c.7806-1G>A* | Intronic | | Causal | [-] 3’SS (MaxEnt: 8,33 → -0,41; NNS: 0,95→<0,4); [+] 3’SS (MaxEnt: -2,51 → 5,54) (1nt downstream) | [-] SC35 (81.13) | [+] hnRNPA1 (73,81) | | ATTCTACTTTTATTTGTTCAAGGCTCTGTGTGACACTCCAG CTGGAGTGTCACACAGAGCCTTGAACAAATAAAAGTAGAAT |
| c.7806-1G>T | Intronic | | Causal | [-] 3’SS (NNS: 0.95 →<0.4); | [-]SC35 (81.13) | [+] 2 PESS;  [-] hnRNPA1 (82,86) | | CTACTTTTATTTGTTCATGGCTCTGTGTGACACTC  GAGTGTCACACAGAGCCATGAACAAATAAAAGTAG |
| c.7806-1_7806-2dup (c.7806insAG) | Intronic | | Causal (frameshift) | MaxEnt: 8.33→6.35 (2 nt downstream)  NNS (=) | [+] SC35 (77.75); [+] 2 SF2 (89.92; 90.74); [+] SRp40 (89.70) | [+] Sironi ESS; [+] hnRNPA1 (86.67) | | TCTACTTTTATTTGTTCAGAGGGCTCTGTGTGACACTCC  GGAGTGTCACACAGAGCCCTCTGAACAAATAAAAGTAGA |
| c.7819A>C | Missense  p.Thr2607Pro | | VUS | - | [-]SF2 (72.31), [-] SC35 (77.63), [-] 2 SRp40 (78.98; 85.27) | [+] 2 Sironi ESS | | TGTTCAGGGCTCTGTGTGACCCTCCAGGTGTGGATCCAAAG  CTTTGGATCCACACCTGGAGGGTCACACAGAGCCCTGAACA |
| c.7875A>G | Synonymous  p.Arg2625= | | VUS | [+] Cryptic 3’SS (NNS: 0.54 → 0.81) | [+] SRp40 (81.74); [-] 2 Rescue-ESE | [-] hnRNPA1 (69.29); [+] Fas-ESS; | | TGGGTTTATAATCACTATAGGTGGATCATATGGAAACTGGC  GCCAGTTTCCATATGATCCACCTATAGTGATTATAAACCCA |
| c.7940T>C | Missense  p.Leu2647Pro | | VUS | - | [-] SRp55 (78.98); [+] 9G8 (61.28) | [-] hnRNPA1 (70.95); [+] Sironi ESS | | GGAATTTGCTAATAGATGCCCAAGCCCAGAAAGGGTGCTTC  GAAGCACCCTTTCTGGGCTTGGGCATCTATTAGCAAATTCC |
| c.7947A>G | Synonymous  p.Pro2649= | | VUS | - | [-]SF2 (77.23), [-] SRp40 (79.40); [-] 2 Rescue-ESE | [-] Sironi ESS | | GCTAATAGATGCCTAAGCCCGGAAAGGGTGCTTCTTCAACT  AGTTGAAGAAGCACCCTTTCCGGGCTTAGGCATCTATTAGC |
| c.7952G>T | Missense  p.Arg2651Met | | VUS | - | [-] SRp40 (79.40); [-] Rescue-ESE | [-] 2 Sironi ESS; [-] Fas-ESS; [+] PESS; [-] hnRNPA1 (83.81) | | TAGATGCCTAAGCCCAGAAATGGTGCTTCTTCAACTAAAAT  ATTTTAGTTGAAGAAGCACCATTTCTGGGCTTAGGCATCTA |
| c.7971A>G | Synonymous  p.Lys2657= | | VUS | - | [-] 2 Tra2-β (81.02; 85.42) | [+] hnRNPA1 (70,48) | | AGGGTGCTTCTTCAACTAAAGTACAGGCAAGTTTAAAGCAT  ATGCTTTAAACTTGCCTGTACTTTAGTTGAAGAAGCACCCT |
| c.7975A>G | Missense  p.Arg2659Gly | | VUS | - | [-]SRp40 (91.86),  [+] SRp55 (75.65);[+] Rescue ESE | [-] hnRNPA1 (74.76) | | CTTCTTCAACTAAAATACGGGCAAGTTTAAAGCATTA  TAATGCTTTAAACTTGCCCGTATTTTAGTTGAAGAAG |
| c.7976G>C | Missense  p.Arg2659Thr | | VUS | [-] GC donor (MaxEnt: 3.1 → 2.4) | [+] 3 SF2 (70.85; 83.84; 80.66); [+] Rescue-ESE | [-] 2 hnRNPA1 (74.76, 66.43) | | TTCTTCAACTAAAATACACGCAAGTTTAAAGCATTAC  GTAATGCTTTAAACTTGCGTGTATTTTAGTTGAAGAA |
| c.7976G>A | Missense  p.Arg2659Lys | | VUS | [-] GC donor (MaxEnt: 3.1 → 2.32) | [+] 2 Rescue-ESE | [-] Sironi ESS; [-] 2 hnRNPA1 (74.76; 66.43) | | GCTTCTTCAACTAAAATACAAGCAAGTTTAAAGCATTACAT  ATGTAATGCTTTAAACTTGCTTGTATTTTAGTTGAAGAAGC |
| c.7976+1G>A | Intronic | | VUS | [-] GC donor (MaxEnt: 3.1 → -5.07) | [-] SRp55 (80.71); [+] 3 SF2 (81.15; 83.58; 74.08); [+] 2 Rescue-ESE; [+] 2 9G8 (61.07; 63.76) | [-] Sironi ESS; [-] hnRNPA1 (74.76) | | TCTTCAACTAAAATACAGACAAGTTTAAAGCATTACA  TGTAATGCTTTAAACTTGTCTGTATTTTAGTTGAAGA |
| c.7976+35C>A | Intronic | | Neutral | - | [-] SRp55(86,98); [-] SF2 (77,46); [-] SF2 (73,44) | [+] hnRNPA1 (74,76) | | TTACATTACGTAATCATATAAGGCAGTATGGTTAAGGTTTC  GAAACCTTAACCATACTGCCTTATATGATTACGTAATGTAA |
| **EXON 18** |  | |  |  |  |  | |  |
| c.7977-119A>T | Intronic | | VUS | [+] 3’SS (NNS: <0.4 → 0.82) | - | [+] 2 PESS | | TTCCTAGCTACAAAATTTTTTATTCTCAGTATTTCTTAGAT  ATCTAAGAAATACTGAGAATAAAAAATTTTGTAGCTAGGAA |
| c.7977-7C>G | Intronic | | VUS | [-] 3’SS (MaxEnt:11.5 →5.7)  [+] 3’SS 6-nt upstream (MAxEnt: 1.1 → 9.2) | [+] SC35 (80.15) | [+] Fas-ESS | | AATATGCATTTTTGTTTTCAGTTTTAGATATGATACGGAAA TTTCCGTATCATATCTAAAACTGAAAACAAAAATGCATATT |
| c.7977-6T>G | Intronic | | VUS | 3’SS NNS: 0,98 → 0,90 |  |  | | ATATGCATTTTTGTTTTCACGTTTAGATATGATACGGAAAT ATTTCCGTATCATATCTAAACGTGAAAACAAAAATGCATAT |
| c.7977-3_7978del | Intronic/exonic deletion | | VUS | [-] 3’SS (NNS: 0,98 → <0,4) | [-] Rescue-ESE | [-] hnRNPA1 (76.90); [-] 2Fas-ESS | | TGCATTTTTGTTTTCACTTTATGATACGGAAATTGATAGA TCTATCAATTTCCGTATCATAAAGTGAAAACAAAAATGCA |
| c.7977-1G>C | Intronic | | VUS | [-] 3’SS (NNS: 0.98→<0.4) | [+]SRp55 (85.90); [-] Rescue ESE | [-] Sironi ESS; [-] 2 Fas-ESS; [+] PESS; [-]hnRNPA1 (76.90) | | CATTTTTGTTTTCACTTTTACATATGATACGGAAATTGATA  TATCAATTTCCGTATCATATGTAAAAGTGAAAACAAAAATG |
| c.7977-2A>T | Intronic | | VUS | [-] 3’SS (NNS: 0,98 → <0,4) |  | [-] hnRNPA1 (76.90); [-] 2Fas-ESS | | GCATTTTTGTTTTCACTTTTTGATATGATACGGAAATTGAT ATCAATTTCCGTATCATATCAAAAAGTGAAAACAAAAATGC |
| c.7977-1G>T | Intronic | | VUS/causal | [-] 3’SS (NNS: 0,98 → <0,4) | [+]SRp55 (75.97) |  | | CATTTTTGTTTTCACTTTTATATATGATACGGAAATTGATA TATCAATTTCCGTATCATATATAAAAGTGAAAACAAAAATG |
| c.7984dupA | Frameshift | | Causal (frameshift) | - | [-] SRp40 (78.98), [-] SF2 (73.09), [-] SRp55 (84.62); [+] 9G8 (75.24) | - | | TTTTCACTTTTAGATATGATAACGGAAATTGATAGAAGCAGA  TCTGCTTCTATCAATTTCCGTTATCATATCTAAAAGTGAAAA |
| c.7985C>G | Missense  p.Thr2662Arg | | VUS | [+] Cryptic 3’SS (NNS: <0.4 → 0.55) | [-] SF2 (73.09), [-] SRp55 (84.62) | [+] 3 Sironi ESS; [+] 2 Fas-ESS | | TCACTTTTAGATATGATAGGGAAATTGATAGAAGCAGAA  TTCTGCTTCTATCAATTTCCCTATCATATCTAAAAGTGA |
| c.7985C>T | Missense  p.Thr2662Met | | VUS | - | [-] SRp40 (78.98), [-] SF2 (73.09); [+] 4 Rescue-ESEs | [+] PESS | | TTTCACTTTTAGATATGATATGGAAATTGATAGAAGCAGAA  TTCTGCTTCTATCAATTTCCATATCATATCTAAAAGTGAAA |
| c.7988A>T | Missense  p.Glu2663Val | | VUS | [+] Cryptic 5’SS (NNS: <0.4 → 0.96) | [-] SRp55 (84.62); [-] PESE | - | | CACTTTTAGATATGATACGGTAATTGATAGAAGCAGAAGATC  GATCTTCTGCTTCTATCAATTACCGTATCATATCTAAAAGTG |
| c.7992T>A | Synonymous  p.Ile2664= | | VUS | - | [-] PESE; [+] Tra2-beta [87.60) | [+] hnRNPA1 (76.90) | | TTTAGATATGATACGGAAATAGATAGAAGCAGAAGATCGG  CCGATCTTCTGCTTCTATCTATTTCCGTATCATATCTAAA |
| c.8007A>G | Synonymous  p.Arg2669= | | VUS | - | [-] 2 Rescue-ESEs; [-] 4 PESE | [+] 2 hnRNPA1 (70.48; 71.19); [+] 2 Sironi ESS | | GAAATTGATAGAAGCAGAAGGTCGGCTATAAAAAAGATAAT  ATTATCTTTTTTATAGCCGACCTTCTGCTTCTATCAATTTC |
| c.8009C>A | Nonsense  p.Ser2670X | | Causal (nonsense) | - | [-] Rescue-ESE; [-] PESE | [+] hnRNPA1 (89.05); [+] Fas-ESS | | AATTGATAGAAGCAGAAGATAGGCTATAAAAAAGATAATGG  CCATTATCTTTTTTATAGCCTATCTTCTGCTTCTATCAATT |
| c.8009C>G | Missense  p.Ser2670Trp | | VUS | - | [-] PESE; [+] 9G8 (64.70) | [+] hnRNPA1 (66.43); [+] 2 Sironi ESS; [+] 2 Fas-ESS; [+] PESS (24.86) | | AATTGATAGAAGCAGAAGATGGGCTATAAAAAAGATAATGG  CCATTATCTTTTTTATAGCCCATCTTCTGCTTCTATCAATT |
| c.8009C>T | Missense  p.Ser2670Leu | | VUS | - | [-] Rescue-ESE; [-] PESE | [+] Sironi ESS | | AATTGATAGAAGCAGAAGATTGGCTATAAAAAAGATAATGG  CCATTATCTTTTTTATAGCCAATCTTCTGCTTCTATCAATT |
| c.8010_8032del | Frameshift | | Causal (frameshift) | - | [-]SC35 (76.09); [-] SRp40 (80.96); [-] 6 Rescue-ESE; [-] 4 Tra2-beta; [-] 2 9G8 | [-] 2 hnRNPA1 (65.48; 84.76); [-] 4 Sironi-ESS; [-] Fas-ESS; [-] PESS | | CGGAAATTGATAGAAGCAGAAGATCGGGATGACACAGCTGCAAAAACACT  AGTGTTTTTGCAGCTGTGTCATCCCGATCTTCTGCTTCTATCAATTTCCG |
| c.8019A>G | Synonymous  p.Lys2673= | | VUS | - | [-] Tra2-beta (83.21); [+] Rescue-ESE; [+] PESE | [+] hnRNPA1 (71.43); [-] PESS | | CAGAAGATCGGCTATAAAGAAGATAATGGAAAGGGATGA  TCATCCCTTTCCATTATCTTCTTTATAGCCGATCTTCTG |
| c.8023A>G | Missense  p.Ile2675Val | | Causal (splicing) | [+] Cryptic 5’SS (NNS: <0.4 →0.92) | [-]SRp40 (80.96); [-] 2 Rescue-ESE; [-] 2 9G8 (65.17; 62.15); [-] Tra2-beta (86.88) | [+] 2 hnRNPA1 (66.67; 73.10); [+] Fas-ESS; [+] PESS | | GAAGATCGGCTATAAAAAAGGTAATGGAAAGGGATGACACA  TGTGTCATCCCTTTCCATTACCTTTTTTATAGCCGATCTTC |
| c.8027T>C | Missense  p.Met2676Thr | | VUS | - | [-] SRp40 (80.96); [-] 3 Rescue-ESE | [-] Sironi ESS; [-] Fas-ESS | | CGGCTATAAAAAAGATAACGGAAAGGGATGACACAGC  GCTGTGTCATCCCTTTCCGTTATCTTTTTTATAGCCG |
| c.8027T>A | Missense  p.Met2676Lys | | VUS | - | [+] 9G8 (61.34) | [+] 2 hnRNPA1 (68.57; 76.43); [-] Fas-ESS | | ATCGGCTATAAAAAAGATAAAGGAAAGGGATGACACAGCTG  CAGCTGTGTCATCCCTTTCCTTTATCTTTTTTATAGCCGAT |
| c.8035G>T | Missense  p.Asp2679Tyr | | Causal (splicing) | [+] Cryptic 5’SS (NNS: <0.4 → 0.96) | - | [+] 2 hnRNPA1 (66.67; 70.48); [-] 2 Sironi ESS;[+] PESS | | GATAATGGAAAGGTATGACACAGCTGC  GCAGCTGTGTCATACCTTTCCATTATC |
| c.8039A>G | Missense  p.Asp2680Gly | | VUS | - | [-]SRp40 (78.98) | [+]hnRNPA1 (67.38) [+] Sironi ESS; [+]Fas-ESS; [+]PESS | | AAAGATAATGGAAAGGGATGGCACAGCTGCAAAAACACTTG  CAAGTGTTTTTGCAGCTGTGCCATCCCTTTCCATTATCTTT |
| c.8042C>G | Missense  p.Thr2681Arg | | VUS | - | - | [+] hnRNPA1 (71.43) | | GGAAAGGGATGACAGAGCTGCAAAAACAC  GTGTTTTTGCAGCTCTGTCATCCCTTTCC |
| c.8072C>T | Missense  p.Ser2691Phe | | VUS | - | [-] 2 SF2 (76.08; 75.01);  [-] SC35 (77.81] | [-] Sironi ESS; [-] Fas-ESS; [+] 2 PESS | | CACTTGTTCTCTGTGTTTTTGACATAATTTCATTGAGC  GCTCAATGAAATTATGTCAAAAACACAGAGAACAAGTG |
| c.8084C>T | Missense  p.Ser2695Leu | | VUS | - | [-] PESE | [+] hnRNPA1 (65.48); [+] 2 PESS | | CTGTGTTTCTGACATAATTTTATTGAGCGCAAATATATCTG  CAGATATATTTGCGCTCAATAAAATTATGTCAGAAACACAG |
| c.8111C>T | Missense  p.Ser2704Phe | | VUS | - | [-] SC35 (78.18);[-] SRp40 (84.79); [-] Rescue-ESE | [-] Sironi ESS | | GCGCAAATATATCTGAAACTTTTAGCAATAAAACTAGTAGTGC  GCACTACTAGTTTTATTGCTAAAAGTTTCAGATATATTTGCGC |
| c.8165C>G | Missense  p.Thr2722Arg | | VUS | - | [-] 2 SF2 (77.62;79.62); [-] SRp40 (83.89); [-] SRp55 (80.71); [+]Rescue-ESE |  | | GGCCATTATTGAACTTAGAGATGGGTGGTATGCTG  CAGCATACCACCCATCTCTAAGTTCAATAATGGCC |
| c.8168A>G | Missense  p.Asp2723Gly | | Causal (missense-splicing) | [+] Cryptic 5’SS (NNS: <0. 4→0.81) | [-] 2 SF2 (77.62; 79.62); [-] Rescue-ESE; [-] 9G8 (64.70) | [-] Sironi ESS; [+] Fas-ESS | | GCCATTATTGAACTTACAGGTGGGTGGTATGCTGTTAAG  CTTAACAGCATACCACCCACCTGTAAGTTCAATAATGGC |
| c.8191C>T | Nonsense  p.Gln2731X | | Causal (nonsense) |  | [-]SF2 (77.23) | [+] hnRNPA1 (73.10); [-] Sironi ESS; [+] Fas-ESS | | GGTGGTATGCTGTTAAGGCCTAGTTAGATCCTCCCCTCTTA  TAAGAGGGGAGGATCTAACTAGGCCTTAACAGCATACCACC |
| c.8219insT | Frameshift  p.Leu2740?fs | | Causal (frameshift) | [+] Cryptic 3’SS (NNS: <0. 4→ 0.44) | [+] SRp40 (79.04) | [+] PESS | | CCTCCCCTCTTAGCTGTCTTTAAAGAATGGCAGACTGAC  GTCAGTCTGCCATTCTTTAAAGACAGCTAAGAGGGGAGG |
| c.8249_8250del | Frameshift  p.Lys2750AsnfsX | | Causal (frameshift) |  | [-] 2 SF2 (80.15; 83.87); [-] SRp40 (86.29); [-] 2 9G8 (86.91; 62.15) [-] Tra2-beta (86.88); [-] 2 Rescue-ESE; [-] 4 PESE | [-] Sironi ESS; [-] Fas-ESS | | CAGACTGACAGTTGGTCAGAATTATTCTTCATGGAGCAGA  TCTGCTCCATGAAGAATAATTCTGACCAACTGTCAGTCTG |
| c.8331G>A | Synonymous  p.Lys2777= | | VUS | [-] 5’SS (NNS: 0.96 →<0.4) | [+]Tra2-beta (83.21) | [-] 3 hnRNPA1 (68.57; 66.67; 73.10); [-] Sironi ESS; [-] Fas-ESS | | CCAGAATCTCTTATGTTAAAAGTAAATTAATTTGCACTCT  AGAGTGCAAATTAATTTACTTTTAACATAAGAGATTCTGG |
| c.8331+1G>T | Intronic | | Causal (splicing) | [-] 5’SS (NNS: 0.96 →<0.4) | [-] SRp40 (88.74) | [-] 2 hnRNPA1 (66.67; 73.10); [-] Sironi ESS; [-] Fas-ESS; [-] PESS | | CAGAATCTCTTATGTTAAAGTTAAATTAATTTGCACTCTTG  CAAGAGTGCAAATTAATTTAACTTTAACATAAGAGATTCTG |
| *c.8331+2T>C* | Intronic | | Causal (splicing) | [-] 5’SS (NNS: 0.96 →<0.4) | [+] 9G8 | [-] Fas-ESS | | AGAATCTCTTATGTTAAAGGCAAATTAATTTGCACTCTTGG  CCAAGAGTGCAAATTAATTTGCCTTTAACATAAGAGATTCT |
| **Artificial mutations** | | **Bioinformatics^2,3,4^** | | | | | **Primers for Site-directed Mutagenesis (5’→3’)** | |
| **Microdeletions^5^** | |  | | | | |  | |
| c.7808_7837del30 | | ^-^ | | | | | ATATTCTACTTTTATTTGTTCAGGGAGCTTATTTCTAGAATTTGGGTTTA  TAAACCCAAATTCTAGAAATAAGCTCCCTGAACAAATAAAAGTAGAATAT | |
| c.7833_7862del30 | | ^-^ | | | | | CAGGGCTCTGTGTGACACTCCAGGTGTGGATAATCACTATAGATGGATCATATGGAAACT  AGTTTCCATATGATCCATCTATAGTGATTATCCACACCTGGAGTGTCACACAGAGCCCTG | |
| c.7919_7948del30 | | ^-^ | | | | | AACTGGCAGCTATGGAATGTGCCTTTCCTAAAAGGGTGCTTCTTCAACTAAAATACAGGC  GCCTGTATTTTAGTTGAAGAAGCACCCTTTTAGGAAAGGCACATTCCATAGCTGCCAGTT | |
| **c.7944_7973del30** | | **ESEs:**: [-] SF2 (IgM-BRCA1) (77.2); 3 SRp40 (79.4, 81.2,91.9); SRp55 (81.4); [-] 3 Rescue-ESEs;[-] 14 PESE; [-] 2 Tra2-β (81, 85.4) | | | | | TCCTAAGGAATTTGCTAATAGATGCCTAAGCAGGCAAGTTTAAAGCATTACATTACGTAA  TTACGTAATGTAATGCTTTAAACTTGCCTGCTTAGGCATCTATTAGCAAATTCCTTAGGA | |
| *c.7944_7953del10* | | **-** | | | | | TCCTAAGGAATTTGCTAATAGATGCCTAAGGTGCTTCTTCAACTAAAATACAGGCAAGTT  AACTTGCCTGTATTTTAGTTGAAGAAGCACCTTAGGCATCTATTAGCAAATTCCTTAGGA | |
| *c.7954_7963del10* | | [-] SRp55 (81.4), SRp40 (81,2); [+] SF2/ASF (73.8); [±] Rescue-ESEs; [+] hnRNPA1 (67.6) | | | | | TTTGCTAATAGATGCCTAAGCCCAGAAAGGAACTAAAATACAGGCAAGTTTAAAGCATTA  TAATGCTTTAAACTTGCCTGTATTTTAGTTCCTTTCTGGGCTTAGGCATCTATTAGCAAA | |
| *c.7964_7973del10* | | [-] SRp40 (91.9); [-] 2 Tra2-β (81, 85.4); [-] 1 Rescue-ESE | | | | | GATGCCTAAGCCCAGAAAGGGTGCTTCTTCCAGGCAAGTTTAAAGCATTACATTACGTAA  TTACGTAATGTAATGCTTTAAACTTGCCTGGAAGAAGCACCCTTTCTGGGCTTAGGCATC | |
| **c.7979_8008del30** | | **ESEs:**: [-] 2 SRp40; (78.98,78.14); SF2 (73.09); SF2 (IgM-BRCA1) (80.15, 83.87); SRp55 (84.62); [-] 10 Rescue-ESEs; [-] 8 PESE; [-] 4 9G8 (60.94, 68.52, 70.74, 86.91), [-] Tra2-β (86.88) | | | | | TGCATTTTTGTTTTCACTTTTAGATCGGCTATAAAAAAGATAATGGAAAG  CTTTCCATTATCTTTTTTATAGCCGATCTAAAAGTGAAAACAAAAATGCA | |
| *c.7979_7988del10* | | **ESEs: SRp40 (78.98) SF2 (73.09); SRp55 (84.62)** | | | | | AAATATGCATTTTTGTTTTCACTTTTAGATAATTGATAGAAGCAGAAGATCGGCTATAAA  TTTATAGCCGATCTTCTGCTTCTATCAATTATCTAAAAGTGAAAACAAAAATGCATATTT | |
| *c7989_7998del10* | | **-** | | | | | TTTTGTTTTCACTTTTAGATATGATACGGAAGCAGAAGATCGGCTATAAAAAAGATAATG  CATTATCTTTTTTATAGCCGATCTTCTGCTTCCGTATCATATCTAAAAGTGAAAACAAAA | |
| *c.7999_8008del10* | | **ESEs: SRp40 (78.14); SF2 (83.87); Tra2-β (86.88)** | | | | | ACTTTTAGATATGATACGGAAATTGATAGACGGCTATAAAAAAGATAATGGAAAGGGATG  CATCCCTTTCCATTATCTTTTTTATAGCCGTCTATCAATTTCCGTATCATATCTAAAAGT | |
| **c.8004_8033del30** | | **ESEs:**:: [-]SF2 (IgM-BRCA1) (80.15), SF2 (83.87), SC35 (76.09), SRp40 (80.96);  [-] 12 Rescue-ESEs; [-] 7 PESE; [-] 4 9G8 (70.74, 86.91, 65.17, 62.15); [-] 5 Tra2-β (86.88, 94.14, 94.14, 83.21, 86.88) | | | | | ATGATACGGAAATTGATAGAAGCAGGGATGACACAGCTGCAAAAACACTT  AAGTGTTTTTGCAGCTGTGTCATCCCTGCTTCTATCAATTTCCGTATCAT | |
| *c.8004_8013del10* | | **ESEs: SF2 (83.87); Tra2-β (86.88); SC35 (76.09)** | | | | | TAGATATGATACGGAAATTGATAGAAGCAGATAAAAAAGATAATGGAAAGGGATGACACA  TGTGTCATCCCTTTCCATTATCTTTTTTATCTGCTTCTATCAATTTCCGTATCATATCTA | |
| *c.8014_8023del10* | | **-** | | | | | ACGGAAATTGATAGAAGCAGAAGATCGGCTTAATGGAAAGGGATGACACAGCTGCAAAAA  TTTTTGCAGCTGTGTCATCCCTTTCCATTAAGCCGATCTTCTGCTTCTATCAATTTCCGT | |
| *c.8024_8033del10* | | **-** | | | | | ATAGAAGCAGAAGATCGGCTATAAAAAAGAGGATGACACAGCTGCAAAAACACTTGTTCT  AGAACAAGTGTTTTTGCAGCTGTGTCATCCTCTTTTTTATAGCCGATCTTCTGCTTCTAT | |
| c.8274_8303del30 | | **-** | | | | | AGATTATTCTTCATGGAGCAGAACTTGAAGCCCCAGAATCTCTTATGTTA  TAACATAAGAGATTCTGGGGCTTCAAGTTCTGCTCCATGAAGAATAATCT | |
| c.8299_8328del30 | | **-** | | | | | GGTGGGCTCTCCTGATGCCTGTACAAAGGTAAATTAATTTGCACTCTTGG  CCAAGAGTGCAAATTAATTTACCTTTGTACAGGCATCAGGAGAGCCCACC | |
| **c.[7984A>T; 8001C>T; 8003G>A]** | | ESE disruptions: SF2 (73.09); SF2 (83.87); SRp40 (78.98); SRp55 (84.62) | | | | | ACTTTTAGATATGATTCGGAAATTGATAGAAGTAAAAGATCGGCTATAAA  TTTATAGCCGATCTTTTACTTCTATCAATTTCCGAATCATATCTAAAAGT | |
| c. 7984A>T | | ESE disruptions: SF2 (73.09); SRp40 (78.98); SRp55 (84.62) | | | | | TTTTCACTTTTAGATATGATTCGGAAATTGATAGAAGCAGA  TCTGCTTCTATCAATTTCCGAATCATATCTAAAAGTGAAAA | |
| c. 8001C>T | | ESE disruptions: SF2 (83.87) | | | | | GATACGGAAATTGATAGAAGTAGAAGATCGGCTATAAAAAA  TTTTTTATAGCCGATCTTCTACTTCTATCAATTTCCGTATC | |
| c. 8003G>A | | ESE disruptions: SF2 (83.87) | | | | | TACGGAAATTGATAGAAGCAAAAGATCGGCTATAAAAAAGA  TCTTTTTTATAGCCGATCTTTTGCTTCTATCAATTTCCGTA | |
| c.7976+21_7976+140del | | **-** | | | | | TAAAATACAGGCAAGTTTAAAGCATTACATATCCCTAGCCCCCATTTAAGAGAGATCACA  TGTGATCTCTCTTAAATGGGGGCTAGGGATATGTAATGCTTTAAACTTGCCTGTATTTTA | |
| c.7976+136_7976+240del | | **-** | | | | | TGACAGTTGCCATCCCACACTGCTGTTCTCATATGAGTTACTAATTTGATCCACTATTTG  CAAATAGTGGATCAAATTAGTAACTCATATGAGAACAGCAGTGTGGGATGGCAACTGTCA | |
| c.7976+231_7977-141del | | ESEs: [-] 3 SRp40; [-] 3 SC35; [-] SF2; [-] SRp55; [-] 4 9G8; [-] 10 Tra2-beta; [-] 10 Rescue-ESE; [-] 3 PESE  ESSs: [-] 4 hnRNPA1; [-] 5 Sironi ESS; [-] 5 Fas-ESS; [-] 18 PESS | | | | | CCACCCCCTCCTTAACCTCTTGATGTATGAATTCCTAGCTACAAAATTTTTAATTCTCAG  CTGAGAATTAAAAATTTTGTAGCTAGGAATTCATACATCAAGAGGTTAAGGAGGGGGTGG | |
| c.7977-150_7977-21del | | **-** | | | | | AAAATAATTGATATTTTAACAATATGAAACATTTTTGTTTTCACTTTTAGATATGATACG  CGTATCATATCTAAAAGTGAAAACAAAAATGTTTCATATTGTTAAAATATCAATTATTTT | |
| c.7976+2C>T | | Switch to a canonical GT site (NNSplice= 1.0) | | | | | TTCTTCAACTAAAATACAGGTAAGTTTAAAGCATTACATTA  TAATGTAATGCTTTAAACTTACCTGTATTTTAGTTGAAGAA | |

1. Spliceogenic variants are highlighted in yellow.
2. HSF: Human Splicing Finder. NNS: NNSplice. [+] and [-] symbols indicate creation or disruption, respectively, of splicing regulatory sequences. The most relevant data are indicated in bold-type.
3. **Cut-offs of the splicing programs:**

***Splice Sites:***

NNSPLICE (values 0-1): Cut-off=0.4 for both 5’ and 3’ splice sites (Reese et al., 1997).

HSF: Human Splicing Finder matrices (default values, cut-offs cannot be defined) (Desmet et al., 2009).

MaxEnt: 0 for 5’ and 3’ splice sites (Yeo and Burge, 2004).

**Enhancers and Silencers:**

ESEfinder cut-offs (HSF scale 0-100): SF2/ASF: 72.98 / SF2/ASF (IgM-BRCA1): 70.51 / SRp40: 78.08 / SC35: 75.05 / SRp55: 73.86 (Cartegni et al., 2003)

ESE motifs from HSF, cut-offs values (0-100): Tra2: 75.964 / 9G8: 59.245

Algorithms Rescue-ESE, Fas-ESS, PESE and PESS predict generation or disruption of motifs with presumed ESE or ESS activity (Fairbrother et al., 2002; Zhang and Chasin, 2004; Wang et al., 2004)

Sironi silencers (Sironi et al., 2004), cut-offs values (0-100): Motif 1: 60 / Motif 2: 60 / Motif 3: 60.

hnRNP motifs: hnRNP A1: 65.476

1. Bioinformatics data of microdeletions with no impact on splicing are not presented, except for the intronic deletion c.7976+231_7977-141del where a high concentration of Tra2-beta motifs were observed.
2. Positive microdeletions and artificial point mutations are shown in red.
