# Supplementary material for: Functional classification of DNA variants by hybrid minigenes: Identification of 30 spliceogenic variants of BRCA2 exons 17 and 18
Source: PLoS Genet. 2017 Mar 24;13(3):e1006691. doi: 10.1371/journal.pgen.1006691 (PMC5384790; doi:10.1371/journal.pgen.1006691)
Supplement: S3 Table — (PDF) [file pgen.1006691.s003.pdf]

S3 Table. Relative quantification of transcripts induced by DNA variants and microdeletions of *BRCA2* exons 17and 18 in MCF-7 cells.

DNA variants	Canonical Transcript[1]	Exon 17 skipping	Ex17-del20	Ex17-del69	Exon17-insAG	Ivs16-ins8	Ex17-del1	Other aberrant transcripts[3]
**EXON 17**								
c.7806-9T>G		**41.5% ±0.3**		22.2% ±0.2		36.3% ±0.5		
c.7806-2A>G		20.1% ±0.3	**51.8% ±0.3**	28.1% ±0.1				
c.7806-1G>A							**100%**	
c.7806-1G>T			**100%**					
c.7806insAG		5.1% ±0.5		2.3% ±0.1	**92.6% ±0.6**			
c.7875A>G	**95.3% ±1.1**			4.7% ±1.1				
c.7975A>G	**73.8% ±0.1**	26.2% ±0.1						
c.7976G>C		**100%)**						
c.7976G>A		**100%**						
c.7976+1G>A		**100%**						
Microdeletions								
c.7944_7973del	**61.6% ±0.2**	38.4 ±0.2						
c.7944_7953del	**100%**							
c.7954_7963del	**97.8%±0.5**	1.0±0.1						1.2%±0.5
c.7964_7973del	**95.4%±0.2**	4.6%±0.2						
**EXON 18**								

DNA variants	Canonical Transcript	Exon 18 skipping	Ex18-ins6	Ex18-del309	Ex18-del298	Ex18-del164	Ex18-del191	Other aberrant transcripts[3]
Wild type Minigene[2]	**99.3 ±0.8**	0.7% ±0.8						
c.7977-7C>G		21.6% ±0.5	**78.4% ±0.5**					
c.7977-6T>G	**66.7% ±0.8**	31% ±0.8					2.3%	
c.7977-3_7978del		**90% ±1.1**					10% ±1.1	
c.7977-2A>T		93.3% ±0.1					6.7% ±0.1	
c.7977-1G>C		**89.8% ±1.8**					10.2% ±1.8	-
c.7977-1G>T		**91.5% ±0.1**					7%±0.1	ex18-del236=1.5%
c.7985C>G		**90.2% ±4.4**					5.0% ±0.1	4.7% ±4.5
c.7985C>T	**96.7 ±1.3**	3.3% ±1.3						
c.7988A>T	**84.2% ±1.5**	8.6% ±1.7						7.2% ± 0.4
c.7992T>A	**68.6% ±1.0**	31.4% ±1.0						
c.8007A>G	**84.8% ±0.6**	15.2%±0.6						
c.8009C>A	4.0 ±3.4	**91.2% ±4.4**					4.8% ±3.9	-
c.8009C>T	**76.6% ±6.1**	23.4% ±6.1						-

c.8009C>G	**79.9 ±1.2**	20.1% ±1.2						
c.8023A>G		0.7% ±0.1		**93% ±0.3**				1.0% ±0.1/ 4.2±0.2
c.8035G>T	2.4% ± 0.5				**93.6% ± 0.4**			4.0±0.2
c.8042C>G	**97.7% ±0.1**	2.3% ±0.1						
c.8072C>T	**94.9% ±1.2**	5.1% ±1.2						
c.8168A>G	**69.6% ±0.9**	4.5% ±0.4				25.9% ±0.5		
c.8249_50del	**93.0 ±1.2**	7.0 ±1.2						
c.8331G>A	40.7% ±0.6	**52.0% ±0.4**						2.1% ±0.1 / 1.7 ±0.1 / 3.5%±0.2
c.8331+1G>T		**81.0 ±0.3**						6.1%±0.2% / 3.7% ±0.3 / 6.4% ±0.4
c.8331+2T>C		**87.1%±0.3**						[ex17-del151 +ex18 skipping] (12.9%)
Microdeletions								
c.7979_8008del	47.2% ±0.4	**49.6% ±0.4**					3.2% ±0.1	
c.7979_7988del	**79.8% ±0.7**	20.2%±0.7						
c.7989_7998del	**100%**							
c.7999_8008del	48.3% ±0.4	**51.7% ±0.4**						
c.8004_8033del	30.6% ±0.2	**64.7% ±0.0**					4.7% ±0.1	
c.8004_8013del	16.3% ±0.1	**78.1% ±0.1**					5.6%	
c.8014_8023del	**100%**							
c.8024_8033del	**100%**							

Relative proportions (%) of each transcript with their standard deviations (±%) are indicated. Principal transcripts are shown in bold type.

[1] Size of the canonical transcript 1012 nt after cDNA amplification with primers RTBR2_ex16-FW and RTpSAD-RV.

[2] The wild type minigenes showed a minor transcript with exon 18 skipping ranging from to 0% to 2.6%.

[3] Other aberrant transcripts: [Ex17del151+Ex18 skipping], [Ex18 sk + ivs17ins58], [Ex18del156], [~710 nt],[775 nt], [822 nt], [867 nt], [878 nt], [1021 nt]
