# Supplementary material for: Functional classification of DNA variants by hybrid minigenes: Identification of 30 spliceogenic variants of BRCA2 exons 17 and 18
Source: PLoS Genet. 2017 Mar 24;13(3):e1006691. doi: 10.1371/journal.pgen.1006691 (PMC5384790; doi:10.1371/journal.pgen.1006691)
Supplement: S4 Table — (PDF) [file pgen.1006691.s004.pdf]

S4 Table. Analysis of the 52 assayed variants according to the guidelines of the American College of Medical Genetics and Genomics (ACMG).

DNA variant[1]	Previous classification	ACMG Criteria[2]	Potential ACMG Classification	# database records
**EXON 17**				
c.7806-40A>G	VUS	PP3, BS3	VUS	28
c.7806-9T>G	VUS	PS3, PM2, PM4, PP3	Likely Pathogenic (SP.)	1
c.7806-2A>G	Causal	PVS1 (SP.), PS3, PM2, PM4, PP3	Pathogenic (SP.)	7
c.7806-1G>A	Causal	PVS1 (SP.), PS3, PM2, PP3	Pathogenic (SP.)	1
c.7806-1G>T	Causal	PVS1 (SP.), PS3, PM2, PP3	Pathogenic (SP.)	1
c.7806-1_7806-2dup	Causal (frameshift)	PVS1 (SP.), PS3, PM2, PP3	Pathogenic (SP.)	2
c.7819A>C	VUS	PM2, PP3, BS3	VUS	1
c.7875A>G	VUS	PM2, PP3, BS3	VUS	2
c.7940T>C	VUS	PM2, PP3, BS3	VUS	6
c.7947A>G	VUS	PP3, BS3	VUS	1
c.7952G>T	VUS	PM2, PP3, BS3	VUS	1
c.7971A>G	VUS	PM2, PP3, BS3	VUS	2
c.7975A>G	VUS	PM2, PP3	VUS	17
c.7976G>C	VUS	PS3, PM2, PP3	Likely Pathogenic (SP.)	2
c.7976G>A	VUS	PS3, PP3, PP5	Likely Pathogenic (SP.)	18
c.7976+1G>A	VUS	PVS1 (SP.), PS3, PM2, PP3	Pathogenic (SP.)	3
c.7976+35C>A	Neutral	PP3, BS3	VUS	16
**EXON 18**				
c.7977-119A>T	VUS	PP3, BS3	VUS	1
c.7977-7C>G	VUS	PS3, PM2, PM4, PP3	Likely Pathogenic (SP.)	4
c.7977-6T>G	VUS	PM2, PP3	VUS	1
c.7977-3_7978del	VUS	PVS1 (SP.), PS3, PM2, PP3	Pathogenic (SP.)	1
c.7977-2A>T	VUS	PVS1 (SP.), PS3, PM2, PP3	Pathogenic (SP.)	1
c.7977-1G>T	VUS	PVS1 (SP.), PS3, PM2, PP3	Pathogenic (SP.)	1
c.7977-1G>C	VUS/causal	PVS1 (SP.), PS3, PM2, PP3	Pathogenic (SP.)	18
c.7984dupA	Causal (frameshift)	PVS1 (Fr.), PM2, PP3	Pathogenic (Fr.)	1
c.7985C>G	VUS	PS3, PM2, PP3	Likely Pathogenic (SP.)	1
c.7985C>T	VUS	PM2, PP3, BS3	VUS	5
c.7988A>T	VUS	PM2, PP3	VUS	9
c.7992T>A	VUS	PP3	VUS	6
c.8007A>G	VUS	PP3	VUS	2
c.8009C>A	Causal (nonsense)	PVS1 (Non.), PS3 (SP.), PM2, PP3	Pathogenic (SP.)	1
c.8009C>G	VUS	PM2, PP3	VUS	6
c.8009C>T	VUS	PM2, PP3	VUS	17
c.8010_8032del	Causal (frameshift)	PVS1 (Fr.), PM2, PP3	Pathogenic (Fr.)	1
c.8019A>G	VUS	PM2, PP3, BS3	VUS	1
c.8023A>G	Causal (splicing)	PS3, PM2, PP3, PP5	Likely Pathogenic (SP.)	5
c.8027T>C	VUS	PP3, BS3	VUS	3
c.8027T>A	VUS	PM2, PP3, BS3	VUS	1
c.8035G>T	Causal (splicing)	PS3,PM2,PP3	Likely Pathogenic (SP.)	2
c.8039A>G	VUS	PM2, PP3, BS3	VUS	2
c.8042C>G	VUS	PM2, PP3, BS3	VUS	1
c.8072C>T	VUS	PM2, PP3	VUS	4
c.8084C>T	VUS	PM2, PP3, BS3	VUS	4
c.8111C>T	VUS	PP3, BS3	VUS	5
c.8165C>G	VUS	PM2, PP3, BS3	VUS	6
c.8168A>G	Causal (missense-splicing)	PS3 (Mis.), PM2, PP3, PP5	Likely Pathogenic (Mis.-SP.)	13
c.8191C>T	Causal (nonsense)	PVS1 (Non.), PM2, PP3	Pathogenic (Non.)	3

c.8219insT	Causal (frameshift)	PVS1 (Fr.), PM2, PP3	Pathogenic (Fr.)	1
c.8249_8250del	Causal (frameshift)	PVS1 (Fr.), PM2, PP3	Pathogenic (Fr.-SP.)	6
c.8331G>A	VUS	PS3, PM2, PP3, PP5	Likely Pathogenic (SP.)	1
c.8331+1G>T	Causal (splicing)	PVS1 (SP.), PS3, PM2	Pathogenic (SP.)	1
c.8331+2T>C	Causal (splicing)	PVS1 (SP.), PS3, PM2	Pathogenic (SP.)	1

[1] **Variants with impact on splicing are yellow-shadowed**

[2] **ACMG criteria used in this study:**

Very strong evidence of pathogenicity

PVS1: null variant (nonsense, frameshift, canonical ±1 or 2 splice sites, initiation codon, single or multiexon deletion) in a gene where LOF is a known mechanism of disease. When this criterion is applied, the type of mutation is indicated between brackets: SP, splicing; Non., nonsense; Fr, frameshift; Mis., missense.

Strong evidence of pathogenicity

PS3: Well-established in vitro or in vivo functional studies (splicing assays) supportive of a damaging effect on the gene or gene product.

Moderate evidence of pathogenicity

PM2: Absent from controls. All variants were checked in the Exome Aggregation Consortium database (exome data from 60,706 unrelated individuals; http://exac.broadinstitute.org/)

PM4: Protein length changes as a result of in-frame deletions/insertions in a nonrepeat region or stop-loss variants (this item has been used for variants that induce in-frame transcripts such as ex18-ins6)

Supporting evidence of pathogenicity

PP3: Multiple lines of computational evidence support a deleterious effect on the gene or gene product. In this study, all variants were checked with NNSplice and Human Splicing Finder 3.0 that contains several splicing algorithm (see also S2 Table).

PP5: Reputable source recently reports variant as pathogenic, but the evidence is not available to the laboratory to perform an independent evaluation (only used for missense, nonsense and frameshift variants).

Strong evidence of benign impact

BS3: Well-established in vitro or in vivo functional studies show no damaging effect on protein function or splicing

Reference: “Guidelines of the American College of Medical Genetics and Genomics (ACMG) “

Richards S, Aziz N, Bale S, Bick D, Das S, Gastier-Foster J, et al. Standards and guidelines for the interpretation of sequence variants: a joint consensus recommendation of the American College of Medical Genetics and Genomics and the Association for Molecular Pathology. Genet Med. ; 2015;17: 405–423. doi:10.1038/gim.2015.30
