# Supplementary material for: Functional classification of DNA variants by hybrid minigenes: Identification of 30 spliceogenic variants of BRCA2 exons 17 and 18
Source: PLoS Genet. 2017 Mar 24;13(3):e1006691. doi: 10.1371/journal.pgen.1006691 (PMC5384790; doi:10.1371/journal.pgen.1006691)
Supplement: S5 Table — (PDF) [file pgen.1006691.s005.pdf]

S5 Table. Distribution of pathogenic/likely pathogenic variants by type according to the BRCA Share database and this study.

	**Pathogenic variants**		**Total (%)**
**Type of mutation**	Exon 17	Exon 18	
**Frameshift**	5	24	29 (44.6%)
**Nonsense**	2	8	10 (15.4%)
**Missense**	1	5	6 (9.2%)
**Splicing**	8	10	**20 (30.8%)**
**TOTAL**	16	47	65
