# Supplementary figures and images for: Functional classification of DNA variants by hybrid minigenes: Identification of 30 spliceogenic variants of BRCA2 exons 17 and 18
Source: PLoS Genet. 2017 Mar 24;13(3):e1006691. doi: 10.1371/journal.pgen.1006691 (PMC5384790; doi:10.1371/journal.pgen.1006691)

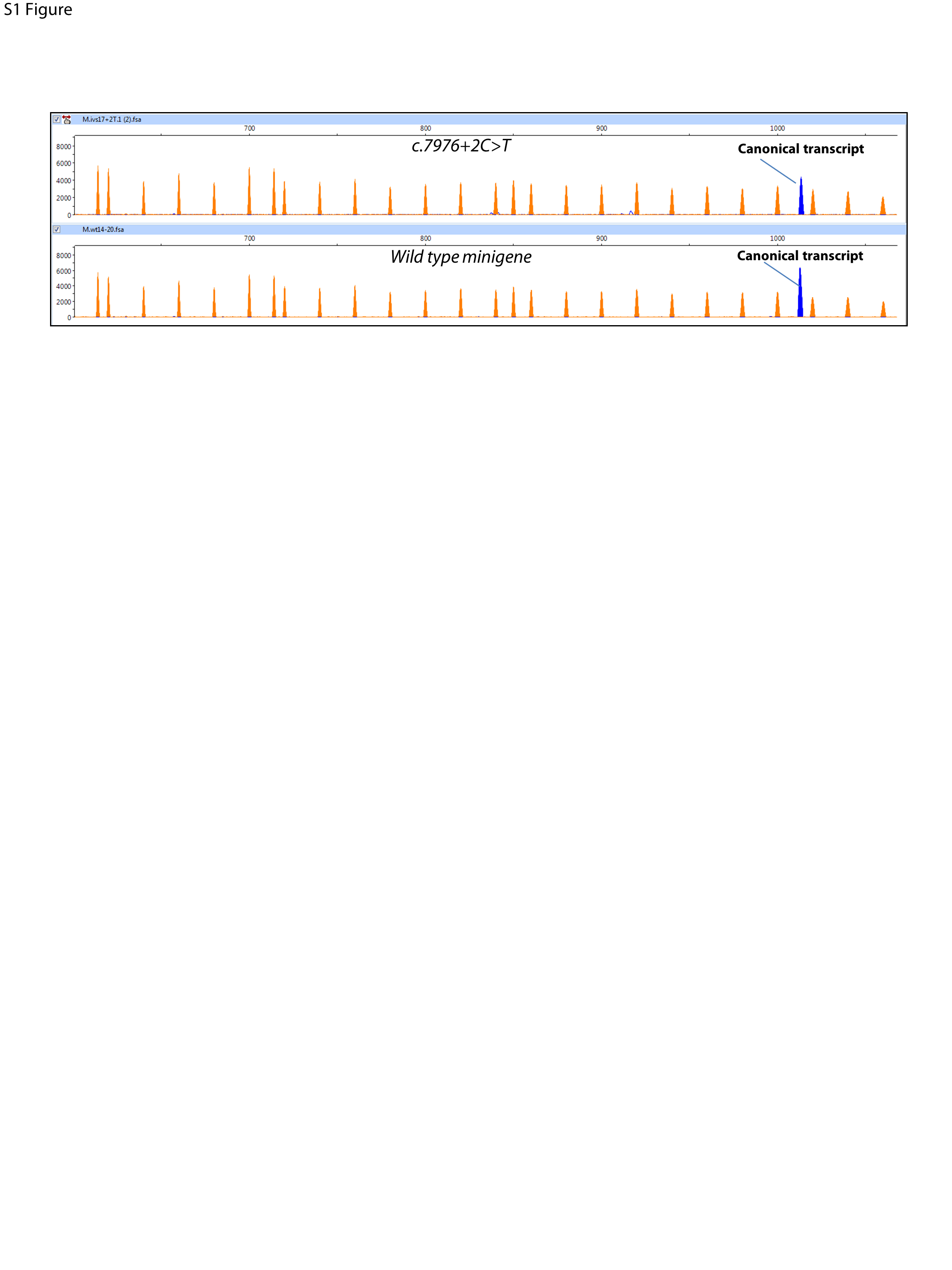

Supplement: S1 Fig — The blue peaks denote the full-length transcripts induced by both minigenes and the Genescan 1200 Size Standard (Applied Biosystems) is shown as orange peaks. (TIF) [file pgen.1006691.s006.tif]

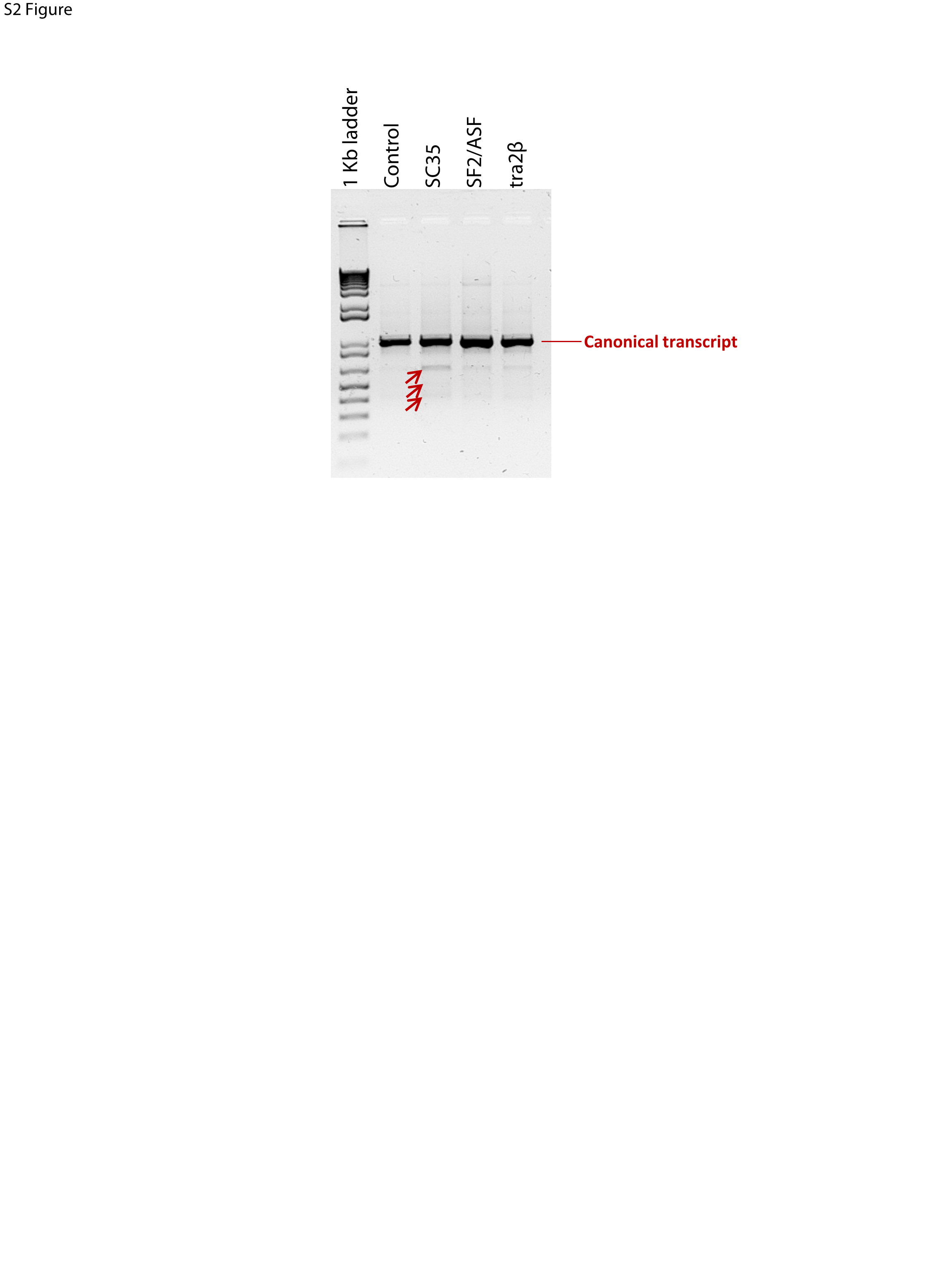

Supplement: S2 Fig — About 1.5x10^5^ MCF7 cells were subjected to a two-hit transfection with Oligofectamine (Thermo Fisher Scientific) and custom-made small interfering RNA (siRNA) at a final concentration of 0.08μM on day 2, the minigene was transfected with Lipofectamine (Thermo Fisher Scientific) on day 4, and RNA was extracted on day 5. The following siRNAs were used: SC35 (aauccaggucgcgaucgaa), SF2/ASF (acgauugccgcaucuacgu), Tra2β (ggaucuucgugaaguauuu) and a control luciferase siRNA. RT-PCR products were run on an agarose gel (1.0%) and stained with Ethidium Bromide. Several aberrant transcripts are visualized, some of which are indicated by arrows. (TIF) [file pgen.1006691.s007.tif]

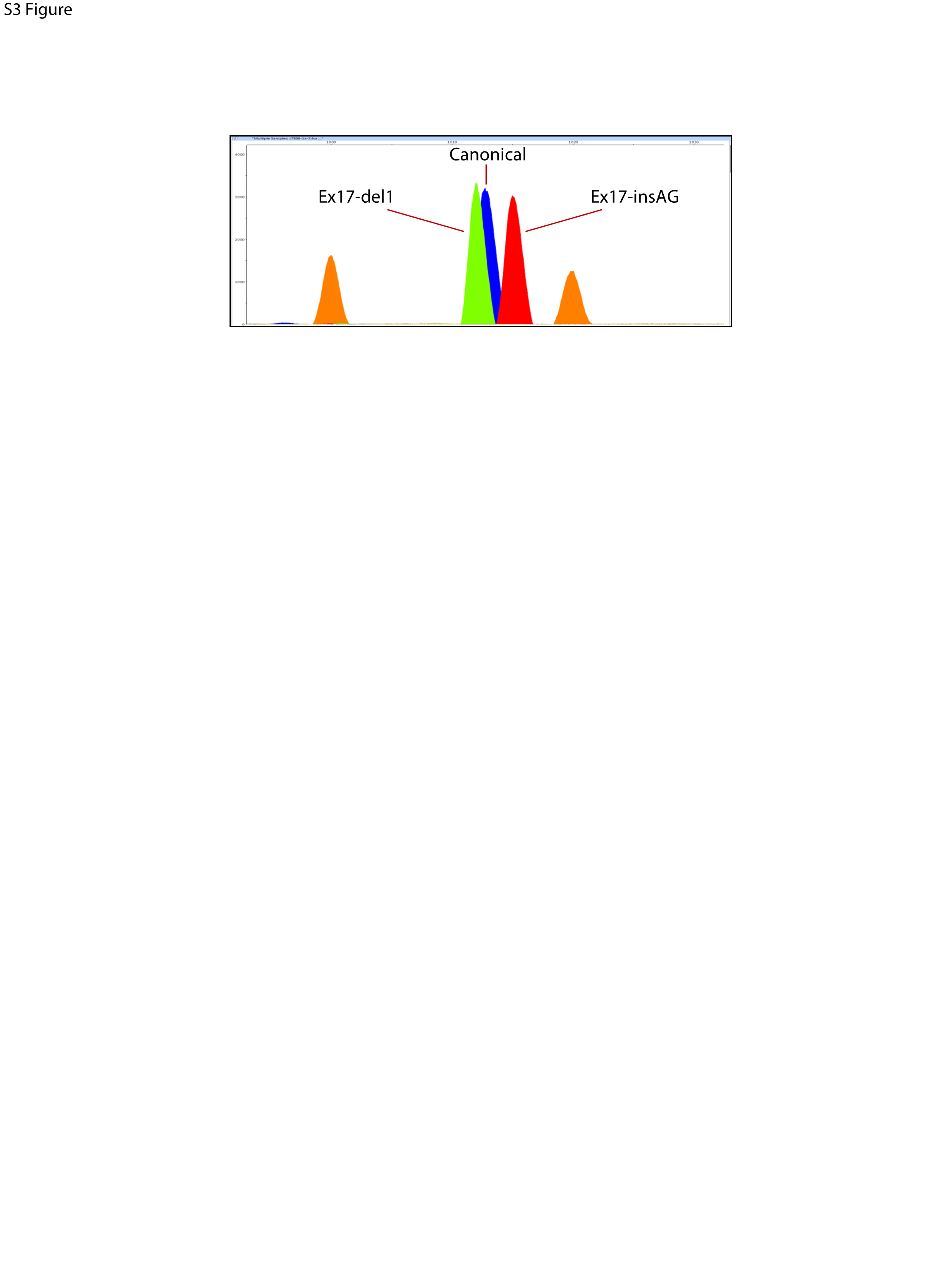

Supplement: S3 Fig — Electropherograms of the wild type minigene and mutations c.7806-1G>A and c.7806-1_7806-2dup were overlaid. Transcripts ex17-del1 and ex17-insAG differed in size by only 1 and 2 nucleotides, respectively, from the canonical transcript despite the large size (1,012 nt) of the RT-PCR products. The Liz-1200 size standard is shown as orange peaks. (TIF) [file pgen.1006691.s008.tif]
